# Investment attractiveness in BRICS+ economies: Evaluating business environment reforms, institutional quality, and macroeconomic factors

**DOI:** 10.1371/journal.pone.0334043

**Published:** 2025-10-16

**Authors:** Mark Awe Tachega, Junjian Wang, Yanjiao Chen, Rizwan Ahmed, Erica Odwira Opoku, Clement Mintah, Leonora Bart-Plange

**Affiliations:** 1 Henan Normal University, College of Political Science and Public Administration, Xinxiang, China; 2 Koforidua Technical University, Koforidua, Ghana; 3 Henan Normal University, College of Social Affairs, Xinxiang, China; 4 Henan Normal University, Research Center for Social Work and Social Governance, Xinxiang, China; 5 School of MBA Shandong University of Finance and Economics, Jinan, China; 6 Taiyuan University of Technology, College of Economics and Management, Taiyuan, China; 7 Koforidua Technical University, Department of Integrated Development Planning, Koforidua, Ghana; 8 International Business University, Toronto, Canada; Tribhuvan University, NEPAL

## Abstract

Investment is a key driver of economic growth in emerging markets, yet the factors that enable or hinder foreign direct investment (FDI) and domestic investment (DI) remain contested. Regulatory reforms aimed at improving the ease of doing business (EDB) have been widely promoted as a means to attract capital, but empirical evidence on their effectiveness, especially in diverse and institutionally heterogeneous economies, is mixed. This study examines how EDB affects investment flows in nine BRICS+ economies (Brazil, Russia, India, China, South Africa, Egypt, Ethiopia, Iran, and the United Arab Emirates) over 2004–2020. Three objectives guide the analysis: assessing the direct EDB-investment link, testing the mediating role of institutional quality, specifically, regulatory quality, government effectiveness, and control of corruption, and analyzing moderation by financial development, economic freedom, and macroeconomic stability. A Pooled Mean Group, Autoregressive Distributed Lag (PMG-ARDL) approach is employed for the panel estimations, complemented by Structural Equation Modelling (SEM) to quantify mediation effects. The results reveal a dual effect of EDB, as it stimulates DI but exerts a modest negative long-term impact on FDI, likely due to transitional adjustment costs and the withdrawal of targeted investment incentives. Mediation analysis shows that institutional quality strongly channels the benefits of EDB to both FDI and DI, with indirect effects often exceeding direct ones. Financial development strengthens the positive EDB-investment relationship, while economic freedom and inflation partially dampen it. Country-specific results indicate substantial heterogeneity in the EDB-investment nexus, with some economies experiencing counterintuitive or insignificant effects. Policy implications differ by investment type. For FDI, reforms should be sequenced gradually, supported by transitional incentives, and aligned with targeted institutional strengthening to offset short-term deterrents. For DI, priority should be given to reducing operational costs, expanding access to finance, and maintaining macroeconomic stability, which collectively enhance domestic firms’ capacity to invest and grow.

## 1. Introduction

Investment serves as the lifeblood of economic growth and development, particularly for emerging economies [1,2]. Beyond immediate financial gains, investment shapes a nation’s trajectory toward long-term prosperity by fueling technological advancements, job creation, and improved living standards [3]. Additionally, investment fosters innovation, enabling developing economies to compete globally and accelerate their growth [4]. Strategic investments in education, healthcare, and community development further empower marginalized populations, promoting inclusive growth and societal well-being [5]. While domestic investment (DI) supports capital formation, infrastructure development, and productive capacity [6], foreign direct investment (FDI) plays a pivotal role in developing countries by bringing not only capital but also managerial expertise and access to international markets [7,8]. The ability of FDI to facilitate technology transfer and knowledge diffusion makes it a critical driver of economic transformation [9].

Despite its importance, identifying the key catalysts of investment remains a challenge for policymakers. Scholars have traditionally examined macroeconomic factors such as infrastructure, trade openness, and financial stability [10]. However, investment decisions are shaped by a complex interplay of factors beyond these fundamentals, encompassing institutional quality, governance efficiency, and regulatory frameworks [11,12]. Strong institutions, political stability, and the rule of law have emerged as critical drivers of investment attractiveness [12–17]. In particular, the ease of doing business (EDB) has become a focal frontier in shaping investment decisions, as market policies that promote transparency, minimize regulatory burdens, and protect intellectual property rights are essential for fostering a pro-business environment [18–21].

The EDB index serves as a crucial measure of a country’s regulatory efficiency, bureaucratic effectiveness, and overall business friendliness, directly influencing both foreign and domestic investment decisions [22,23]. A well-structured regulatory framework ensures stability, predictability, and lower investment risks, making economies more attractive to capital inflows [24]. Empirical evidence suggests that business-friendly environments, characterized by streamlined regulatory processes, contract enforcement, and efficient tax administration, are positively correlated with higher investment inflows [25,26]. These elements collectively shape an economy’s ability to attract and sustain long-term investment, ultimately catalyzing economic growth [2,20,27].

The BRICS economies Brazil, Russia, India, China, and South Africa, including the newly-admitted nations (Egypt, Ethiopia, Iran, and the United Arab Emirates) (In 2024, the BRICS group expanded to include Egypt, Ethiopia, Iran, and the United Arab Emirates, reflecting broader regional and economic diversification. While these new members were formally admitted in 2024, their investment climates and business regulations have been evolving independently for decades. Given the availability of historical data, this study includes both the founding and newly admitted BRICS members in its empirical analysis, covering the period from 2004 to 2020. For brevity, the term BRICS+ is used throughout this study to refer collectively to the original five BRICS economies along with the four newly admitted members.), herein referred to as BRICS+ , are significant players in the global economic landscape due to their economic scale, resource endowments, and strategic positioning in emerging markets [28,29]. Collectively, they account for over 30% of global GDP and attract approximately 20% of global foreign direct investment inflows, underscoring their significance in shaping international capital movements [30]. As some of the largest and fastest-growing markets, these nations offer substantial investment opportunities due to their economic dynamism, expanding consumer markets, and evolving regulatory landscapes, making them attractive to foreign and domestic investors.

In response to the growing importance of attracting investment, BRICS+ nations have initiated major business environment reforms aimed at improving regulatory efficiency and reducing bureaucratic barriers. Several member countries have implemented targeted policy changes to streamline their frameworks. For instance, India has undertaken comprehensive tax digitalization to enhance transparency and efficiency [31], while China has simplified business registration procedures to accelerate market entry [32,33]. Brazil has adopted deregulation strategies to reduce compliance burdens, facilitating smoother operations for both domestic and foreign enterprises [34]. Collectively, these reforms reflect broader efforts by BRICS+ economies to position themselves as competitive and attractive destinations for sustainable capital inflows.

Beyond individual country-level efforts, BRICS+ nations have also pursued collaborative strategies to strengthen their investment environment. The establishment of the New Development Bank (NDB) represents a strategic move toward alternative investment financing, reducing reliance on traditional Western financial institutions and fostering greater financial autonomy among emerging markets [35,36]. However, despite these initiatives, progress in improving EDB across BRICS+ remains uneven. Many of these economies continue to grapple with persistent challenges, including regulatory inefficiencies, bureaucratic bottlenecks, and governance disparities, all of which impact their ability to attract and sustain investment [37].

While previous research has established a positive correlation between ease of doing business and foreign direct investment inflows [19,38–41], the mechanisms through which business environment reforms translate into tangible investment outcomes remain underexplored. Specifically, the extent to which institutional factors, regulatory quality, governance effectiveness, and corruption control mediate this relationship is underexplored. Moreover, the moderating role of macroeconomic conditions, economic stability, financial development, and economic freedom remains an open question. This study seeks to bridge these gaps by empirically analyzing how EDB influences investment flows in BRICS economies while accounting for both institutional mediation and macroeconomic moderation. By offering a more nuanced understanding of these interactions, the study provides critical insights for policymakers seeking to foster a business-friendly investment climate that supports sustainable economic growth.

Therefore, this study makes three key contributions to the literature. First, while existing research has established a general link between ease of doing business (EDB) and foreign direct investment (FDI), this study expands the scope by also examining its impact on domestic investment, an often-overlooked area in investment research. Second, unlike prior studies that primarily assess EDB’s direct effects, this research incorporates institutional mediation, specifically regulatory quality, governance effectiveness, and corruption control, to provide a deeper understanding of how business environment reforms translate into investment outcomes. Third, this study introduces a macroeconomic moderation framework, evaluating how financial development, economic stability, and economic freedom shape the EDB-investment relationship. Additionally, by conducting a country-specific analysis, this research moves beyond treating BRICS+ as a homogenous entity, offering a more granular understanding of investment dynamics within individual member states. By including both founding and newly admitted BRICS+ members, this study presents a more inclusive assessment of investment attractiveness in the evolving BRICS+ landscape, providing empirical insights that can guide investment policy and regulatory reforms in emerging markets.

The rest of this paper is structured as follows: Section 2 reviews the relevant literature. The methodology employed in this study is detailed in Section 3. Section 4 presents the empirical findings and discusses the results. Finally, Section 5 concludes the paper and puts forth policy recommendations.

## 2 Literature review

### 2.1 Theoretical literature

The theoretical underpinnings of investment are rooted in various theories that attempt to explain the motives, determinants, and patterns of cross-border investment flows. Dunning [42] investment development path (IDP) theory suggests that a country’s FDI evolves with its economic development, transitioning from attracting inward FDI due to location advantages to generating outward FDI as domestic firms develop ownership advantages. The location-based approach complements this by focusing on how firm-specific behaviors interact with a host country’s economic geography, such as resource endowment, market size, and government policies. Whether resource-seeking, market-seeking, efficiency-seeking, or strategic asset-seeking, firm motives interplay with these locational determinants [43]. Notably, the institutional theory by North [44] emphasized the role of well-structured institutions in reducing uncertainty, safeguarding property rights, and fostering a stable investment environment. The quality of institutions significantly impacts investment decisions by shaping economic incentives and activities.

Closely related, the Governance Regulation Theory (GRT) focuses on the design, implementation, and enforcement of regulatory frameworks as a central determinant of economic performance and investment attractiveness. It posits that transparent, predictable, and efficiently administered regulations reduce transaction costs, mitigate risks of opportunism, and enhance market confidence [45,46]. Within the GRT framework, regulatory quality is viewed as a governance function that interacts with broader institutional settings to influence investor perceptions and decisions. Weak regulatory governance, manifested in inconsistent enforcement, excessive bureaucracy, or legal ambiguity, can erode the benefits of formal business reforms, whereas strong governance amplifies the effectiveness of such reforms. This theoretical lens is central to the present study, as it underpins the hypothesized mediating role of institutional quality in translating regulatory reforms, measured through the EDB index, into tangible investment outcomes in BRICS+ economies.

Building on this, the social influence theory of the EDB by Doshi et al. [47] suggests that a friendly business environment creates a snowball effect that guides investors’ decisions, attracting investment inflows. In contrast, the Keynesian economic theory underscores the importance of interest rates and income levels in determining investment, with lower interest rates and higher incomes stimulating investment by increasing expected returns and available capital [48]. These theories highlight the interplay between a country’s economic development stage, locational advantages, institutional framework, business environment, and macroeconomic conditions in shaping FDI patterns.

### 2.2 Empirical review

#### 2.2.1 The impact of EDB on foreign direct investment (FDI).

FDI is a critical driver of economic growth, particularly in emerging economies, as it facilitates capital inflows, technology transfer, and market integration [49]. The EDB index has been widely recognized as a determinant of FDI, as it reflects the regulatory efficiency, business-friendly policies, and institutional stability that reduce transaction costs and enhance investor confidence [50] . Empirical studies consistently indicate that higher EDB rankings correlate with increased FDI inflows, particularly in economies that historically faced regulatory barriers [51]. Regulatory simplifications, trade liberalization, and institutional reforms have been linked to FDI growth in BRICS economies [52]. A comparative analysis between BRICS and G7 economies found that BRICS nations experienced a higher compounded annual growth rate in FDI, indicating that regulatory efficiency plays a central role in shaping investor decisions [53]. Cross-country research further supports this, demonstrating that improved EDB rankings are significantly associated with greater FDI inflows, particularly in emerging markets where cumbersome regulations have historically hindered investment [54].

Within BRICS+ economies, China has consistently attracted the highest FDI inflows, supported by sustained regulatory reforms. Its focus on reducing bureaucratic inefficiencies, simplifying business registration, and expanding special economic zones has enhanced its investment appeal. Empirical evidence suggests that China’s improvements in investor protection and contract enforcement have played a key role in maintaining its position as a global FDI leader [52]. India has also implemented substantial EDB reforms, including the introduction of the Goods and Services Tax (GST) and digitalization of business registration and compliance processes, contributing to an improved EDB ranking and increased FDI inflows [37]. However, the impact of EDB reforms on FDI is not uniform across BRICS+ economies. In Russia, domestic regulatory improvements have not fully translated into sustained FDI growth due to external geopolitical risks and economic sanctions, which overshadow business climate reforms [55]. South Africa similarly faces challenges despite its relatively stable EDB ranking, as policy uncertainty, infrastructure deficits, and regulatory inefficiencies remain deterrents for investors. Brazil has made incremental improvements to its business environment, yet bureaucratic complexity and tax burdens continue to constrain FDI inflows. Research comparing business reforms in Latin America and BRICS countries suggests that while Brazil has streamlined investment procedures, regulatory fragmentation remains a key obstacle [37].

Among the newly admitted BRICS+ members, the United Arab Emirates (UAE) stands out as a leading investment hub due to its highly efficient regulatory framework and pro-business policies. Streamlined business registration systems, tax incentives, and strong contract enforcement mechanisms have made it one of the most attractive FDI destinations [56]. However, not all EDB dimensions exert equal influence on FDI attraction. Studies in Southeast Asia demonstrate that contract enforcement, minority investor protection, and tax efficiency are key determinants of FDI, whereas indicators such as ease of access to credit may have weaker or even negative effects in some contexts [57]. While EDB reforms generally encourage FDI, their impact varies by economic context. Earlier studies suggested that in highly developed economies, marginal changes in EDB rankings may not significantly influence FDI, as these nations already maintain efficient business environments. However, recent evidence indicates that the effect of EDB reforms is most pronounced in emerging markets, where regulatory inefficiencies have historically deterred investment. A 130-country analysis found that in developing economies, an improved EDB score was associated with significantly greater FDI inflows at a 1% significance level, while in developed economies, the relationship was statistically insignificant [58]. These findings suggest that in economies like BRICS+, reducing regulatory barriers can substantially enhance investor confidence and capital inflows, whereas in already well-regulated economies, investment decisions are influenced by other factors. Research across various regions confirms the significance of EDB in attracting FDI. Studies in Eastern Africa [59,60], Sub-Saharan Africa [20,61], and West Africa [18] all show that simplifying business procedures and improving tax administration are crucial for attracting investment. Factors like access to credit, security, and market size also impact FDI [59]. Furthermore, studies indicate that positive governance and institutional indicators, such as control of corruption and political stability, also play a crucial role in fostering investment [38].

Despite strong empirical support for the positive relationship between EDB and FDI, EDB reforms alone are insufficient for sustaining investment growth. Several studies emphasize that business-friendly regulatory frameworks must be complemented by macroeconomic stability, institutional quality, and governance effectiveness to maximize their impact [38,62]. The experience of BRICS+ economies demonstrates that while EDB reforms facilitate investment, broader economic and institutional conditions ultimately determine whether these investments are sustained over time.

#### 2.2.2 The role of EDB in domestic investment (DI).

The EDB index significantly influences domestic investment by shaping the regulatory environment in which local businesses operate. A favorable EDB framework reduces bureaucratic hurdles, lowers transaction costs, and enhances the overall business climate, encouraging domestic enterprises to expand. Empirical studies confirm a positive correlation between improved EDB rankings and higher levels of DI, particularly in developing economies where regulatory inefficiencies have historically deterred investment [19]. Comparative analyses highlight the varying impact of EDB on DI across BRICS nations. Russia’s higher EDB ranking, attributed to streamlined business registration processes and efficient regulatory frameworks, has fostered a more conducive environment for domestic investment. In contrast, South Africa and Brazil, with weaker EDB performance, have faced challenges in creating business-friendly environments, potentially hindering DI growth [37]. India’s business environment reforms, including the implementation of the Goods and Services Tax (GST), aimed to simplify tax structures and reduce compliance burdens. However, persistent bureaucratic complexities and inconsistent policy enforcement have tempered the anticipated gains, limiting the direct impact on DI [18]. China presents a more successful case, where sustained regulatory reforms, administrative streamlining, and efficiency enhancements have fostered domestic investment growth. The government’s efforts to remove unnecessary regulatory barriers have created a stable and predictable business environment, encouraging long-term capital formation and private-sector expansion [18]. South Africa’s experience, however, illustrates that EDB improvements alone are insufficient to drive DI growth. Despite targeted regulatory reforms, policy uncertainty and infrastructure deficits continue to deter domestic investors, emphasizing the need for broader economic stability and institutional support [18]. While EDB reforms play a crucial role in stimulating domestic investment, they are not standalone solutions. The experiences of BRICS nations indicate that regulatory improvements must be complemented by stable macroeconomic policies, robust institutional frameworks, and targeted initiatives addressing domestic investment constraints to maximize their effectiveness.

#### 2.2.3 Institutional quality and investment attraction.

Institutional quality plays a pivotal role in shaping a country’s attractiveness to both foreign and domestic investors. Governance effectiveness, regulatory quality, political stability, and control of corruption are critical determinants of investment decisions. Empirical research consistently highlights the positive relationship between strong institutional frameworks and FDI inflows. A study on South Asian and Southeast Asian economies found that improved institutional quality, particularly political stability and regulatory effectiveness, significantly boosted FDI [63]. Similarly, research on BRICS nations using a Bayesian time-varying coefficient model over two decades (2000–2022) suggests that while no long-term equilibrium exists between FDI, institutional quality, and economic growth, short-term improvements in governance can lead to immediate increases in FDI [64]. Conversely, weak institutional frameworks deter investment. An analysis of BRICS economies from 1992 to 2019 revealed that geopolitical risks and deteriorating institutional quality negatively impact FDI inflows, with lower rule of law and political instability reducing foreign investments [65]. A meta-analysis of FDI originating from BRICS countries suggests that firms in weak institutional environments often adapt by leveraging their experience in unstable conditions when investing in similarly structured economies. While this indicates resilience, it also highlights the challenges of attracting high-quality, long-term investment when institutional weaknesses persist [66].

Corruption is one of the most well-documented barriers to investment. It functions as an informal “tax” on business, undermines the rule of law, and deters foreign investors. A study of 15 Southern African Development Community (SADC) countries found that higher levels of corruption significantly reduced FDI inflows, with investors preferring jurisdictions that ensure transparency and predictability [67]. Governments that have implemented anti-corruption reforms such as e-governance initiatives and independent oversight agencies tend to attract more investment, as these measures enhance institutional credibility and investor confidence.

Beyond corruption, broader governance indicators, including political stability and policy predictability, are essential for investment attraction. Investors value environments where government policies remain stable over time, as frequent turnover or civil unrest heightens perceived risk. Research on investor behavior confirms that political uncertainty discourages long-term capital commitments, while economies with predictable governance structures tend to sustain higher investment levels [22]. A study on Asia-Pacific economies further demonstrated that political stability, when combined with trade openness, amplifies FDI inflows by providing a secure and competitive market environment [68].

The legal and regulatory framework also emerges as a decisive factor in investment decisions. According to an Atlantic Council analysis, the strength of a country’s legal institutions, including property rights protection, judicial efficiency, and contract enforcement, has the strongest positive association with FDI among governance indicators [22]. Investors require assurance that legal disputes will be resolved fairly and that their assets will be protected under a transparent legal system. Estonia, for example, leveraged comprehensive legal reforms to transform itself into a regional investment hub, demonstrating how institutional improvements can directly enhance economic competitiveness [69]. By contrast, economies with weak legal protections and inconsistent enforcement struggle to retain investor trust, making capital inflows volatile and unpredictable [70].

Institutional quality and EDB reforms are deeply interconnected. While EDB measures focus on streamlining regulatory procedures, institutional strength ensures the effectiveness and credibility of these reforms. Studies show that bureaucratic quality amplifies the benefits of regulatory reforms by ensuring their proper enforcement. In South Asia, for example, an efficient bureaucracy was found to significantly reduce transaction costs, thereby strengthening FDI inflows [71]. The consensus in the literature is that successful investment climates require both strong institutions and regulatory efficiency[72]. A country that is easy to do business in but lacks institutional stability may still struggle to attract and retain investment, while economies that combine business-friendly regulations with transparent governance structures are the most successful in securing long-term investment.

A critical review of the existing literature reveals significant gaps in research on the relationship between EDB and investment. Most studies primarily examine the direct relationship between EDB and FDI [13,19,21,73]. While these studies provide valuable insights, they overlook domestic investment and alternative capital inflows, limiting the understanding of how business environment reforms influence investment at both local and international levels. This narrow scope presents gaps in designing comprehensive economic policies that account for both domestic and foreign capital formation.

Beyond the investment types considered, existing research largely fails to examine the institutional and macroeconomic factors that mediate or moderate the relationship between EDB and investment. Elements such as governance quality, political stability, financial infrastructure, and regulatory enforcement likely influence how EDB reforms translate into actual investment inflows. By focusing primarily on direct causality, previous studies may oversimplify investment determinants, missing the complex mechanisms through which business-friendly policies shape investment decisions.

Another limitation concerns the geographic scope of past research. Most existing studies focus on the five founding BRICS members, with limited attention to the newly admitted nations, Egypt, Ethiopia, Iran, and the United Arab Emirates. Given their diverse economic structures, regulatory frameworks, and governance models, these new members introduce unique investment dynamics that remain underexplored. This study extends the scope by including these new BRICS members and conducting a country-specific analysis, ensuring a more comprehensive assessment of investment dynamics across the expanded group.

To address these gaps, this study provides a broader and more nuanced analysis of investment attractiveness within BRICS+ nations. Unlike prior research that primarily focuses on FDI, this study evaluates both foreign and domestic investment inflows, recognizing the role of local capital formation in economic development. Additionally, it incorporates institutional mediation and macroeconomic moderation into the EDB investment framework, offering a more detailed examination of how governance, stability, and financial conditions shape investment outcomes. By capturing these complexities, this study contributes both theoretically and empirically to advancing research on business environment reforms and investment policies in emerging economies.

## 3 Hypotheses development

The ease of doing business framework is widely recognized as a critical determinant of investment, influencing both FDI and domestic investment by reducing regulatory barriers, improving transparency, and fostering a competitive business climate. Business-friendly regulations create an environment where firms can operate efficiently, reducing transaction costs and mitigating investment risks [18,20]. The EDB index is measured using the World Bank’s composite EDB index, which aggregates ten dimensions of the regulatory environment into a score from 0 to 100, with higher scores indicating a more business-friendly climate. The index directly captures the procedural and regulatory conditions that can influence both foreign and domestic investment flows. Given its role in facilitating investment, we propose:

***Hypothesis 1:*** *A positive relationship exists between EDB and investment.*

To extend this analysis, we consider the role of regulatory quality as a mediating mechanism linking EDB reforms to investment outcomes. Although closely related to institutional quality, regulatory quality is conceptually narrower. Regulatory quality is defined, in line with the World Bank’s Worldwide Governance Indicators, as the government’s capacity to formulate and implement sound policies and regulations that foster private sector development. Institutional quality, by contrast, is a broader construct encompassing regulatory quality alongside governance dimensions such as government effectiveness, control of corruption, political stability, and the rule of law.

Regulatory quality is operationalized using the Worldwide Governance Indicators (WGI) “Regulatory Quality” indicator (–2.5 to 2.5 scale), chosen for its global coverage, comparability across countries, and its direct conceptual alignment with the EDB’s emphasis on regulatory efficiency and policy formulation. A transparent and well-structured regulatory framework reduces policy uncertainty, enhances legal protections, and ensures fair market competition, making an economy more attractive to investors [74]. Countries with strong regulatory institutions offer efficient enforcement mechanisms, streamlined administrative processes, and well-defined property rights, minimizing risks for both foreign and domestic investors [22,63,75,76]. However, EDB reforms alone do not automatically translate into an improved investment climate unless regulatory quality is sufficiently strong to ensure consistent implementation of business-friendly policies. Weak regulatory institutions, characterized by inconsistent enforcement, excessive red tape, and ambiguous legal frameworks, may limit the ability of EDB reforms to generate significant investment inflows. Without strong regulatory quality, EDB reforms remain ineffective in addressing deeper institutional barriers to investment. Given this, we propose:

***Hypothesis 2:*** *Regulatory quality positively mediates the EDB–investment relationship*

Beyond regulatory quality, governance effectiveness is crucial in ensuring that EDB-driven reforms translate into meaningful investment attraction. Governance effectiveness reflects a government’s capacity to design, implement, and enforce policies efficiently, ensuring stability and institutional credibility. Here, governance effectiveness is measured using the WGI “Government Effectiveness” indicator (–2.5 to 2.5 scale), selected because it captures bureaucratic quality, policy consistency, and public service delivery, factors shown in prior research to be essential for translating regulatory reforms into investment growth. Higher EDB rankings often correlate with better governance, yet EDB reforms alone do not guarantee increased investment unless accompanied by adequate state capacity and policy execution [47,77]. Stable governance fosters investor confidence by ensuring consistent policymaking, effective contract enforcement, and the protection of property rights, thereby creating a predictable and secure environment for business activity [78,79] . Conversely, weak governance, manifested in policy reversals, bureaucratic inefficiency, and the absence of institutional accountability, can undermine the intended benefits of EDB reforms. Given this, we propose:

***Hypothesis 3:*** *Governance effectiveness positively mediates the EDB-investment relationship*

Another critical institutional dimension influencing the efficacy of EDB reforms is control of corruption, also measured by the WGI (–2.5 to +2.5 scale). Corruption distorts market mechanisms, undermines fair competition, increases transaction costs, and erodes investor confidence. Even in environments where business regulations are formally simplified, pervasive corruption can neutralise the intended benefits of reform by introducing informal barriers, unpredictability, and rent-seeking behaviours [80,81]. A high EDB ranking does not inherently translate into an investment-friendly environment if corruption remains widespread, as investors may still encounter bribery, favoritism, and opaque regulatory enforcement, undermining the effectiveness of business-friendly policies [82,83]. This measure is employed because it directly reflects the integrity and impartiality of the institutional environment, conditions that are indispensable for translating regulatory reforms into actual investment flows. Countries with robust anti-corruption mechanisms foster a more transparent, fair, and competitive business climate, allowing investors to operate with greater certainty regarding contract enforcement, market-entry, and operational efficiency [84,85]. On the other hand, in environments where corruption is entrenched, even well-designed EDB reforms may be rendered ineffective, as informal costs and bribery negate the intended benefits of streamlined regulations. When corruption is well controlled, EDB-driven regulatory improvements are more likely to translate into real investment gains, as businesses trust that legal protections and policies will be enforced fairly. Thus, corruption control is a critical institutional filter that determines whether EDB reforms successfully translate into increased investment. Therefore, we propose:

***Hypothesis 4:*** *Control of corruption positively mediates the EDB-investment relationship*

Beyond institutional quality, macroeconomic conditions play a crucial role in shaping the EDB-investment relationship by influencing the extent to which business-friendly regulatory reforms translate into real economic gains. Even when business environments are favorable, investment outcomes can vary depending on the strength of financial markets, economic freedom, and macroeconomic stability. Financial development, measured here as domestic credit to the private sector (% of GDP), enhances the benefits of EDB reforms by improving access to finance and reducing borrowing costs. In economies with well-developed financial systems, businesses can readily secure funding, allowing them to capitalize on regulatory improvements and expand operations [86,87] . In such environments, investment inflows tend to be more responsive to EDB-driven reforms, as financial institutions facilitate the movement of capital and support long-term business expansion [88]. Pal and Mahalik [87] further demonstrate that regulatory reforms in economies with deep financial markets are more likely to translate into sustained investment growth as firms benefit from reduced credit constraints and enhanced financial intermediation. Conversely, in economies with weak financial development, the benefits of EDB reforms may remain unrealized as businesses face liquidity shortages and high borrowing costs that limit their ability to expand despite improved regulatory conditions. This highlights the importance of financial sector development in reinforcing the impact of EDB reforms on investment attraction. As a result, we hypothesize:

***Hypothesis 5:*** *Financial development strengthens the positive relationship between EDB and investment.*

Similarly, economic freedom, measured by the Heritage Foundation’s Economic Freedom Index (0–100 scale, higher values are equal to greater freedom), can amplify the effects of EDB reforms by promoting competition and reducing excessive state intervention. In economies with high economic freedom, businesses operate with greater flexibility, reduced bureaucratic constraints, and stronger legal protections, ensuring that regulatory improvements lead to real economic gains [89]. In this study, financial development is operationalised as the ratio of domestic credit provided by the financial sector to gross domestic product (GDP), following established empirical practice. Empirical studies confirm that the impact of EDB reforms is more pronounced in countries with higher economic freedom. Giovanis and Ozdamar [90] found that firms experience stronger investment responses to business climate improvements in liberalized economies as they operate under transparent and predictable market conditions. Additionally, Capelleras et al. [91] demonstrated that entrepreneurial activity and investment inflows increase significantly in free-market economies following regulatory reforms, reinforcing the role of economic liberalization in maximizing the benefits of EDB. Also, a recent study examining the relationship between financial development and business growth in sub-Saharan Africa found that financial development plays a crucial role in business expansion, suggesting that a robust financial system amplifies the positive effects of EDB reforms on investment [92]. In contrast, EDB reforms may have limited effectiveness in economies with excessive government control, frequent policy reversals, or trade restrictions, as businesses remain uncertain about the long-term regulatory environment [93]. This highlights the importance of a free-market structure in ensuring that EDB-driven reforms successfully stimulate investment. As a result, we hypothesize:

***Hypothesis 6:*** *Economic freedom positively moderates the EDB index-investment relationship.*

Lastly, economic stability, proxied by the annual percentage change in consumer prices (inflation), is a crucial determinant of how effectively EDB reforms translate into sustained investment growth and investor confidence. Investors are more likely to respond positively to business-friendly regulations when inflation is stable, fiscal policies are predictable, and exchange rate volatility is minimized. In contrast, high inflation, fiscal imbalances, and monetary instability introduce uncertainty, reducing the attractiveness of investment opportunities even in economies with strong regulatory frameworks [94]. Research shows that EDB reforms generate stronger investment inflows in stable macroeconomic environments. Kayamba [95] found that investment is more responsive to regulatory improvements in economies with low inflation and sound monetary policies, as investors can make long-term commitments with lower risk. Similarly, Das and Ordal [96] argued that macroeconomic stability enhances the effectiveness of ease of doing business reforms by minimizing financial uncertainty, thereby making long-term capital allocation more attractive. Conversely, even significant regulatory reforms may not yield expected investment growth in economies with high inflation or fiscal unpredictability, as firms delay or scale down expansion plans due to uncertainty. Huang et al. [97] supported this argument, noting that investment responsiveness to business reforms weakens significantly in volatile macroeconomic environments as firms hedge against future financial instability. Economic stability is operationalised using the annual percentage change in the consumer price index (CPI), a standard measure of inflation. This proxy is employed because price stability is a direct and observable indicator of macroeconomic predictability and is strongly associated with the overall stability of the investment climate. By using this measure, the analysis captures the degree to which predictable macroeconomic conditions can reinforce the positive effects of EDB reforms on investment flows:

***Hypothesis 7:*** *Economic stability positively moderates the EDB index-investment relationship.*

## 4. Methodology

### 4.1 Data description and sources

This study uses data from the World Bank Indicators [106], Worldwide Governance Indicators [107], and Heritage Foundation [108] databases, covering the period from 2004 to 2020. This temporal scope was selected because, following the discontinuation of the EDB index in 2021, no directly comparable updates exist that would allow for extending the data without introducing structural breaks or compromising comparability. Extending the dataset beyond 2020 would have introduced methodological inconsistencies and pandemic-related distortions unrelated to the structural relationships examined in this study. The data sources provide comprehensive information on macroeconomic, institutional, and regulatory indicators for the BRICS+ countries. The definitions of variables and the list of countries included in the analysis are presented in S1 and S2 Tables, respectively. Additional information on data construction is provided in the supplementary materials. Table 1 outlines the full set of variables used in the study. While these data sources are authoritative and widely used in empirical research, they are not without limitations. Several governance indicators, such as regulatory quality, government effectiveness, and control of corruption, are perception-based, which may introduce subjective bias and affect cross-country comparability. The Ease of Doing Business index, although comprehensive, was discontinued by the World Bank in 2021 due to concerns over data integrity, raising questions about the consistency of earlier data. Additionally, some time series contain gaps that were addressed through interpolation. These limitations are acknowledged and considered in the interpretation of results.

**Table 1:** Variables.

Indicator	Symbol	Unit	Source
Dependent Variables			
Foreign Direct Investment	FDI	% of GDP	World Bank [109]
Domestic Investment	DI	Gross Capital Formation % of GDP	World Bank [109]
Independent Variables			
Composite ease of doing business	EDB	Aggregate Score	World Bank [106]
Ease of doing business Indicators			
Starting a Business	SB	Score	World Bank [106]
Dealing with Construction Permits	DCP	Score	World Bank [106]
Getting Electricity	GEL	Score	World Bank [106]
Registering Property	RP	Score	World Bank [106]
Getting Credit	GC	Score	World Bank [106]
Protecting Minority Investors	PMI	Score	World Bank [106]
Paying Taxes	PT	Score	World Bank [106]
Trading Across Borders	TAB	Score	World Bank [106]
Enforcing Contracts	EC	Score	World Bank [106]
Resolving Insolvency	RI	Score	World Bank [106]
Control Variables			
Market Size	GDP	Per capita GDP	World Bank [109]
Trade Openness	TRA	% of GDP	World Bank [109]
Exchange Rate	XC	LCU per US$	World Bank [109]
Mediating Variables			
Regulatory Quality	RQ	Estimate	World Bank [107]
Governance Effectiveness	GE	Estimate	World Bank [107]
Control of Corruption	CC	Estimate	World Bank [107]
Moderating Variables			
Financial development	FID	Domestic credit provided by financial sector (% of GDP)	World Bank [109]
Economic Freedom Index	EFI	Index	The Heritage Foundation [108]
Economic Stability	ES	Inflation, consumer prices (annual %)	WorldBank [109]

The institutional mediation variables, Regulatory Quality (RQ), Government Effectiveness (GE), and Control of Corruption (CC) are drawn from the World Bank’s Worldwide Governance Indicators (WGI) database. Each index ranges from approximately –2.5 (weak performance) to +2.5 (strong performance) and is based on composite measures of perceptions derived from a range of surveys and expert assessments. RQ captures perceptions of the government’s capacity to formulate and implement sound policies and regulations conducive to private sector development. GE reflects perceptions of the quality of public services, the competence of the civil service, the quality of policy formulation and implementation, and the credibility of governmental commitments. CC measures perceptions of the extent to which public power is exercised for private gain, including petty and grand forms of corruption as well as state capture. These indicators are used because they directly capture institutional capacities and constraints that may transmit the effects of EDB reforms to investment outcomes, thereby aligning with the mediation hypotheses in Section 3.

Regrading the macroeconomic moderation variables, Financial Development (FID) is measured as the ratio of domestic credit provided by the financial sector to GDP, following established practice in the finance–growth literature [110]. This ratio reflects the depth and efficiency of financial intermediation available to the private sector. Economic Freedom (EFI) is obtained from the Heritage Foundation’s Economic Freedom Index, which scores countries from 0 to 100 across four pillars: rule of law, government size, regulatory efficiency, and market openness. This measure is selected because it provides a comprehensive, internationally comparable assessment of market-oriented policy settings that can shape the effectiveness of regulatory reforms. Economic Stability (ES) is proxied by the annual percentage change in the consumer price index (CPI), a standard measure of inflation widely used to capture price stability and macroeconomic predictability. These moderators are included to assess the extent to which macro-level economic conditions can amplify or constrain the investment effects of EDB reforms, consistent with the theoretical propositions advanced in Section 3.

### 4.2 Empirical strategy

The econometric analysis is conducted in the following sequence: Firstly, descriptive statistics and a Pearson correlation matrix are utilized to examine the data series’ fundamental characteristics and pairwise relationships. Secondly, to assess the impact of common shocks and determine suitable estimation methods, cross-sectional-dependency (CSD) tests are employed, including Breusch and Pagan [100] CDLM1, Pesaran [101] scaled CDLM2, and Pesaran CD tests. Thirdly, Hashem Pesaran and Yamagata [102] slope homogeneity test is performed to check for uniformity of slopes across different units in the panel data. Fourthly, the Variance Inflation Factor (VIF) checks for multicollinearity among predictors in the regression model. Fifthly, the stationarity of the variables is verified using the second-generation Cross-sectionally Im, Pesaran, and Shin (CIPS) developed by [103]. Sixthly, for long-term relationship analysis among variables, Westerlund panel cointegration tests [111] are employed to manage cross-sectional dependency. Seventhly, the PMG-ARDL method estimates dynamic short- and long-term coefficients. Lastly, mediation and moderation analyses are employed to understand how EDB influences investment.

### 4.3 Empirical model

Investment decisions, particularly FDI and domestic investment, are influenced by multiple economic, institutional, and regulatory factors. Moosa [98] highlights the absence of a universally agreed-upon set of FDI determinants, necessitating a multidimensional approach. This study draws on institutional and government regulation theory, which emphasizes the role of legal, administrative, and regulatory frameworks in shaping investment climates, and the Location-Based Approach to FDI Theory highlights the importance of country-specific advantages in attracting foreign and domestic investment. By integrating these perspectives, the study examines how the EDB index, as a measure of regulatory efficiency and market accessibility, influences investment dynamics in BRICS+ economies. The model captures resource-seeking behavior by including natural resource rent (NRT), reflecting the attractiveness of resource-rich economies. Market-seeking behavior is accounted for through GDP (market size) and trade openness (TRA), which indicate accessibility to global markets. Efficiency-seeking behavior is reflected in the EDB index. Additionally, specific EDB indicators are included to provide deeper insights into investment decision-making.

The EDB index captures the overall regulatory efficiency of a country, while its indicators provide insights into specific business climate constraints that may influence investment behavior. Therefore, two separate empirical models are estimated: one that examines the overall impact of the composite EDB index on investment and another that disaggregates the index into its indicators to assess their distinct contributions. Both models incorporate key macroeconomic control variables, including market size, trade openness, natural resource rent, and exchange rate fluctuations, to account for broader economic influences. The general functional form of the empirical model is specified as follows:


$$INV_{it}=\alpha +\beta_{1}EDB_{it}+\beta_{2}GDP_{it}+\beta_{3}TRA_{it}+\beta_{4}NRT_{it}+\beta_{5}XC_{it}+\mu_{i}+\varepsilon_{it}$$ (1)

Where $INV_{it}$ represents investment, disaggregated into FDI and domestic investment; $EDB_{it}$ is the composite ease of doing business score; $GDP_{it}$ accounts for market size; $TRA_{it}$ captures trade openness; $NRT_{it}$ represents natural resource rent; and $XC_{it}$ denotes exchange rate stability. The term $\mu_{i}$ controls for country-specific fixed effects, while $\epsilon_{it}$ is the error term.

To further assess which specific regulatory dimensions exert the strongest influence on investment, the second model decomposes the EDB index into its ten individual indicators:


$$INV_{it}=\alpha +j=\sum_{j=1}^{10}\gamma_{j}EDB_{jit}+\beta_{\text{2}}GDP_{it}+\beta_{3}TRA_{it}+\beta_{4}NRT_{it}+\beta_{5}XC_{it}+\mu_{i}+\varepsilon_{it}$$ (2)

Where $EDB_{jit}$ represents each of the ten ease of doing business indicators, including starting a business (SB), dealing with construction permits (DCP), getting electricity (GEL), registering property (RP), getting credit (GC), protecting minority investors (PMI), paying taxes (PT), trading across borders (TAB), enforcing contracts (EC), and resolving insolvency (RI). Each coefficient $\gamma_{j}$ measures the effect of a specific regulatory dimension on investment, while control variables remain consistent with the first model to allow for comparability.

### 4.4 Cross-sectional dependence and slope homogeneity tests

The presence of cross-sectional dependence (CSD) in panel data can significantly affect the validity of estimations, especially in studies involving multiple countries where economic and institutional interlinkages are strong. This study conducts CSD tests to determine whether economic shocks, policy changes, or external factors in one BRICS country influence others. Ignoring cross-sectional dependence can lead to biased and inconsistent estimates, necessitating second-generation panel estimation techniques. The Pesaran [99] CD test is employed to assess cross-sectional dependence, which is suitable for large panels with a moderate time dimension. The test statistic is given by:


$$CD=\frac{\sqrt{2T}}{N(N-1)}\sum_{i=1}^{N-1}\sum_{j=i+1}^{N}\hat{\rho_{ij}}$$ (3)

Where ${\hat{\rho }}_{ij}$ represents the correlation coefficient of the residuals from different cross-sections. A significant test statistic (CD) suggests that cross-sectional dependence is present, justifying the use of econometric models that account for such dependencies. In addition to the Pesaran CD test, the Breusch and Pagan [100] LM test and Pesaran [101] scaled LM test are performed to confirm the presence of dependence. The Breusch-Pagan LM test statistic is expressed as:


$$LM=\sum_{i=1}^{N=1}\sum_{j=i+1}^{N}T{{\hat{\rho }}^{2}}_{ij}$$ (4)

Where T denotes the time dimension of the panel. For robustness, the Pesaran-scaled LM test adjusts for the bias of the standard LM test in large panels and is computed as:


$$LM_{adj}=\frac{\text{1}}{N(N-1)}\sum_{i=1}^{N=1}\sum_{j=i=1}^{N}\left(\sqrt{T}{\hat{p}}_{ij}-\frac{\text{1}}{T-1}\right)$$ (5)

A rejection of the null hypothesis in these tests indicates that economic linkages among BRICS countries are significant, requiring panel models that account for cross-sectional dependence.

Apart from cross-sectional dependence, slope homogeneity is tested to determine whether the estimated coefficients are consistent across different countries. If slopes are homogeneous, pooled panel estimators can be applied; otherwise, heterogeneous estimators such as PMG-ARDL are more appropriate. The Hashem Pesaran and Yamagata [102] slope homogeneity test is used to assess this, with the test statistic given by:


$$\Delta \tilde{Z}=\frac{\sum {\mathrm{\backslash nolimits}}_{i=1}^{N}{\left(\hat{\beta_{i}}-\overset{―}{\beta }\right)}^{2}}{{\sigma^{2}}_{\hat{\beta_{i}}}}$$ (6)

where ${\hat{\beta }}_{i}$ represents country-specific coefficient estimates, $\bar{\beta }$ is the mean coefficient across all countries, and $\sigma_{{\hat{\beta }}_{i}}^{2}$ denotes the variance of the slope estimates. A statistically significant test statistic suggests that investment behavior varies across BRICS countries, reinforcing the need for an estimation technique accommodating slope heterogeneity. These tests provide the necessary statistical foundation for selecting appropriate panel estimation techniques. If cross-sectional dependence and slope heterogeneity are confirmed, pooled estimators such as Fixed Effects or Random Effects would be unsuitable, necessitating methods such as PMG-ARDL, which allow for heterogeneous short-run dynamics while maintaining homogenous long-run relationships across countries.

### 4.5 Unit root and panel cointegration tests

To ensure robust and consistent parameter estimation, it is essential to test for the stationarity of the variables and establish the presence of a long-run equilibrium relationship between EDB, investment, and macroeconomic controls. This is achieved through a two-stage process, testing for unit roots and testing for cointegration among the non-stationary variables. Given the likelihood of cross-sectional dependence in BRICS+ economies, the study employs the second-generation CIPS unit root test developed by Pesaran [103]. This test augments the standard ADF regression with cross-sectional averages to correct for interdependencies across units. It is specified as:


$$\Delta Y_{it}=\alpha_{i}+\beta_{i}Y_{it-1}+{\gamma_{i}}_{{\overset{―}{Y}}_{t-1}}+\sum_{j=1}^{\rho }\delta_{ij}\Delta Y_{it-j}+\sum_{j=1}^{\rho }\phi_{ij}\Delta {\overset{―}{Y}}_{t-j}+\varepsilon_{it}$$ (7)

where $Y_{it}$ is any of the panel variables (investment, EDB, or control variables), and ${\bar{Y}}_{t}$ denotes the cross-sectional average. The null hypothesis of a unit root is tested against the alternative of stationarity, allowing for heterogeneity in autoregressive coefficients. Following this, the study employs the Westerlund [104] Error Correction-Based Panel Cointegration test, which is specifically designed to detect cointegration by testing for the existence of error correction at the individual and panel levels. Unlike residual-based approaches, the Westerlund ECM test does not require pre-estimated long-run relationships and is robust to cross-sectional dependence. The test is based on the following panel error correction model:


$$\begin{array}{c} \Delta Y_{it}=\alpha_{i}+\phi_{i}(Y_{it-1}-\beta_{i}\prime X_{it-1})+\sum_{j=1}^{\rho }\gamma_{ij}\Delta Y_{it-j}+\sum_{j=0}^{q}\delta_{ij}\Delta X_{it-j}+\varepsilon_{it} \end{array}$$ (8)

where $\phi_{i}$ is the error correction term, and a significant negative value indicates the presence of cointegration. The null hypothesis of no cointegration (i.e., $\phi_{i}=0$) is tested against the alternative that at least one panel unit (or all units) exhibits a restoring force toward long-run equilibrium. The Westerlund test produces four test statistics including Gt, Ga, Pt, and Pa, each serving a different purpose. The Gt and Ga statistics are based on group-mean estimators, allowing for heterogeneity in the cointegration relationship across countries. In contrast, the Pt and Pa statistics are based on pooled estimators and assume some degree of homogeneity. Specifically, Gt and Ga test the null hypothesis of no cointegration for each cross-sectional unit, accommodating heterogeneity, while Pt and Pa test the null hypothesis of no cointegration for the panel as a whole, assuming partial homogeneity. The rejection of the null hypothesis using these statistics provides evidence of cointegration either at the group level (Gt, Ga) or for the panel as a whole (Pt, Pa), supporting the validity of long-run relationships and justifying the subsequent use of the PMG-ARDL estimation framework.

### 4.6 PMG-ARDL model specification

Given the results from the unit root and cointegration tests, which establish the presence of a stable long-run relationship among EDB, investment, and macroeconomic controls, the Pooled Mean Group – Autoregressive Distributed Lag (PMG-ARDL) model is employed as the primary estimation technique. The PMG-ARDL approach, introduced by Pesaran et al. [105], is well-suited for panel data where short-run dynamics may vary across countries, but long-run relationships remain homogenous. This model allows for a more flexible estimation strategy, accommodating heterogeneous short-run adjustments while imposing long-run equilibrium constraints across BRICS economies. The PMG-ARDL model is specified as follows:


$$INV_{it}=\alpha_{i}+\sum_{j=1}^{\rho }\lambda_{ij}INV_{i,t-j}+\sum_{j=0}^{q}\delta_{ij}X_{i,t-j}+\mu_{i}+\varepsilon_{it}$$ (9)

where: $INV_{it}$ represents investment, which is estimated separately for FDI and DI, $X_{it}$ includes the explanatory variables, the aggregate EDB index in Model 1, and the Individual EDB indicators in Model 2. $\lambda_{ij}\;and\;\delta_{ij}$ are coefficients capturing long-run and short-run relationships, respectively. $\mu_{i}$ represents country-specific fixed effects, controlling for unobserved heterogeneity. $\varepsilon_{it}$ is the error term, assumed to be normally distributed. A key advantage of the PMG-ARDL model is its ability to distinguish between short-run and long-run dynamics. While country-specific factors may influence short-run investment behavior, BRICS+ economies are expected to share common long-run equilibrium relationships driven by structural economic and regulatory conditions. To explicitly capture the speed of adjustment toward long-run equilibrium, the PMG-ARDL model incorporates an Error Correction Mechanism (ECM), formulated as:


$$\Delta INV_{it}=\lambda (INV_{it-1}-\theta X_{it-1})+\sum \gamma_{j}\Delta X_{it-j}+\mu_{it}$$ (10)

Where: λ represents the error correction term, which measures the speed at which investment returns to equilibrium following economic shocks. A negative and statistically significant λ confirms the existence of a stable long-run equilibrium. The term $\theta X_{it-1}$ represents the estimated long-run coefficients of the explanatory variables.

PMG-ARDL is chosen over alternative estimation techniques for several reasons. Unlike Fixed Effects or Random Effects models, which assume homogeneous slope coefficients, PMG-ARDL allows for heterogeneous short-run adjustments while ensuring long-run homogeneity, making it ideal for structurally diverse economies such as BRICS+. Compared to Dynamic Panel GMM, which is effective in addressing endogeneity but focuses primarily on short-term dynamics, PMG-ARDL is more appropriate for assessing long-run investment determinants. Furthermore, Fully Modified OLS (FMOLS) and Dynamic OLS (DOLS), while suitable for panel cointegration, assume strictly non-stationary variables, whereas PMG-ARDL accommodates both I(0) and I(1) series without requiring first differencing for stationarity. The application of PMG-ARDL ensures that the estimated relationships between EDB, institutional quality, and investment are both statistically robust and economically meaningful, accounting for country-specific short-run variations while maintaining a shared long-run trajectory across BRICS economies. The optimal lag lengths for the PMG-ARDL models were selected using the Akaike Information Criterion (AIC), with a maximum of three lags allowed for both dependent and independent variables. The selected lag structures generally indicated one lag for the dependent variable and one lag for each regressor, ensuring a consistent specification across all model estimations.

Finally, potential endogeneity concerns, arising from reverse causality, omitted variables, or measurement error, are mitigated by the dynamic nature of PMG-ARDL. The inclusion of lagged dependent and independent variables reduces simultaneity bias, while the ECM framework accounts for long-run equilibrium behaviour. This structure enables robust estimation of both short-run adjustments and long-run relationships between EDB, institutional quality, and investment.

### 4.7 Mediation model

This model employs a structural equation modeling (SEM) approach in the sense of regression-based path analysis to investigate the indirect effects of the EDB index on investment through three mediators: Regulatory Quality (RQ), Government Effectiveness (GE), and Corruption Control (CC). The analysis uses observed variables and Sobel test estimates of indirect effects, together with ratio measures (RIT and RID), to assess the relative importance of mediation pathways, rather than a latent-variable SEM with unobserved constructs. The EDB index, as the independent variable, directly influences each mediator:


$$RQ=\beta_{\text{1}}*EDB+\epsilon_{\text{1}}$$

$$GE=\beta_{\text{2}}*EDB+\mu_{\text{2}}$$ (11)

$$CC=\beta_{3}*EDB+\varsigma_{3}$$

Each mediator and the dependent variables are affected by the EDB index:


$$INV_{it}=\beta_{\text{4}}*EDB+\beta_{\text{5}}*RQ+\beta_{\text{6}}*GE+\beta_{\text{7}}*CC+\varpi_{\text{4}}$$ (12)

To evaluate the indirect effects of EDB on investment via each mediator, the relevant path coefficients are multiplied:


$$EBD\to RQ\to FDI(DI)(Indirect\;effect=\beta_{\text{1}}*\beta_{\text{5}})$$

$$EBD\to GE\to FDI(DI)(Indirect\;effect=\beta_{2}*\beta_{\text{6}})$$ (13)

$$EBD\to CC\to FDI(DI)(Indirect\;effect=\beta_{3}*\beta_{7})$$

The total effect of the EDB index on economic growth is the sum of the combined direct effects $(\beta_{\text{4}})$.

**Fig 1:**
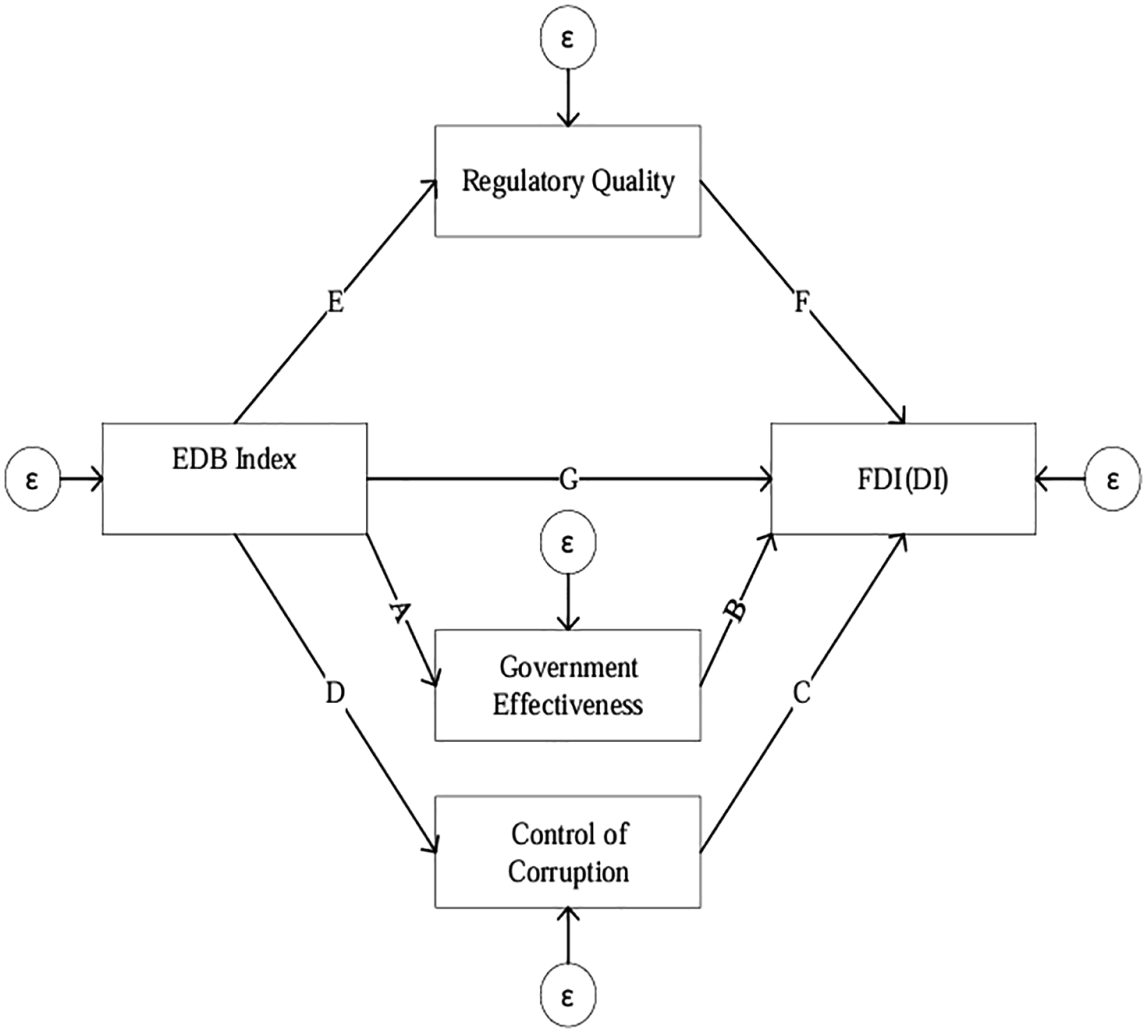
Path analysis diagram of the mediation model.

Where: β: represents path coefficients (strength and direction of the relationships); $\beta_{1}\;\beta_{\text{2}}\;and\;\beta_{\text{3}}$: indicates the direct effect of EDB on each mediator. A positive coefficient suggests EDB improves the mediator, while a negative coefficient suggests it hinders it. $\beta_{4}$: represents the direct effect of EDB on investment, independent of the mediators. $\beta_{\text{5}}\;\beta_{\text{6}}\;and\;\beta_{7}$: indicate the direct effect of each mediator on investment. Positive coefficients suggest a positive influence, while negative coefficients suggest a negative influence. ε,μ,ς,and ϖ: represents error terms (unexplained variance in each equation). Fig 1 illustrates the schematic representation of the mediation framework.

### 4.8 Moderation analysis

In addition to exploring the direct and mediated effects of the ease of doing business (EDB) on investment, this study investigates whether the strength or direction of this relationship varies across different macroeconomic contexts. This is achieved through moderation analysis, which tests whether specific moderating variables condition the impact of EDB on investment. Moderation analysis is implemented by introducing interaction terms between EDB and each moderator in the investment regression model. The model is specified as:


$$INV_{it}=\alpha +\beta_{1}EDB_{it}+\beta_{2}MOD_{it}+\beta_{3}(EDB_{it}\times MOD_{it})+\gamma Z_{it}+\mu_{i}+\varepsilon_{it}$$ (14)

Where, $INV_{it}$ is the investment variable (FDI or DI). $MOD_{it}$ is the moderating variable (FID, ES, or EFI). $\left(EDB_{it}\times MOD_{it})(EDB_{it}\times MOD_{it})(EDB_{it}\times MOD_{it}\right)$ is the interaction term testing whether the effect of EDB varies with the level of the moderator. $Z_{it}$ is a vector of control variables (GDP, TRA, NRT, XC). $\mu_{i}$ captures country-specific effects, and $\varepsilon_{it}$ is the error term. The coefficient $\beta_{3}$ on the interaction term is of particular interest: A positive and significant $\beta_{3}$ suggests that the moderator amplifies the effect of EDB on investment. A negative and significant $\beta_{3}$ implies that the moderator dampens or weakens the EDB-investment relationship. The results provide critical insights into the contextual dependencies of EDB reforms and the policy complementarities required to maximize their investment-inducing effects.

## 5. Empirical results

### 5.1 Descriptive statistics

The preliminary analysis shows significant differences in investment, institutional quality, EDB, and economic variables across the sampled countries. The pairwise correlation analysis reveals strong interconnections between economic, institutional, and financial indicators, emphasizing robust relationships. However, diagnostic tests indicate cross-sectional dependence and slope heterogeneity, necessitating second-generation estimation techniques. Additionally, the unit root test results suggest that while some variables are stationary at levels I(0), most exhibit non-stationarity at levels but become stationary after first differencing, implying integration of order one I(1). The presence of a mix of I(0) and I(1) variables justifies the use of the PMG-ARDL estimator, which is specifically designed to accommodate such combinations of integration orders. Importantly, cointegration tests provide evidence of long-run equilibrium relationships between investment and the explanatory variables. For detailed results, refer to S3–S8 Tables in the supplementary documents. These include descriptive statistics, correlation analysis, cross-sectional dependence test outcomes, Variance Inflation Factor analysis, unit root estimates, and Westerlund cointegration tests. Additionally, S1 File figures present an exploration of average Ease of Doing Business (EDB) indicator values.

### 5.2 Trend analysis of EDB, FDI, and domestic investment

**Fig 2:**
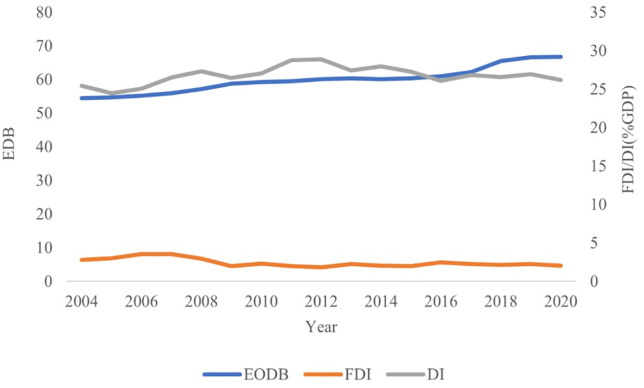
Aggregate trends of EDB, FDI, and DI across BRICS+ Countries (2004–2020). Source: Authors’ calculations based on World Bank data.

To contextualize the investment dynamics explored in the econometric analysis, Fig 2 presents the aggregate trends in the EDB index, FDI, and Domestic Investment (DI) across BRICS+ countries from 2004 to 2020. The EDB index displays a steady upward trajectory, reflecting ongoing regulatory reforms to enhance the business environment across the bloc. However, this regulatory improvement does not correspond with a similar rise in FDI inflows, which remain relatively flat and volatile throughout the period. In contrast, domestic investment is consistently higher and more stable, suggesting a closer alignment between EDB improvements and internal capital formation. These diverging patterns support the hypothesis that while business environment reforms are necessary, their effects on FDI may be delayed or diluted by other structural factors, such as policy credibility, investor sentiment, and institutional quality. In contrast, domestic investors may be more immediately responsive to procedural improvements. These dynamics are explored further in the following econometric results and country-specific analyses.

### 5.3 Short- and long-run coefficient estimates

#### 5.3.1 The impact of the ease of doing business index on FDI and domestic investment.

Prior to estimation, the optimal lag structure for all PMG–ARDL specifications was selected using the Akaike Information Criterion (AIC), with a maximum of three lags permitted for both dependent and independent variables, given the annual data spanning 2004–2020. For the aggregate EDB–investment models (Model 1), the preferred specification was ARDL(1,1,1,1,1,1), denoting one lag for the dependent variable and one lag for each regressor. The EDB-indicator models (Model 2), incorporating all ten indicators within a single specification, adopted an ARDL(1,1,…,1) configuration, corresponding to one lag for the dependent variable and one lag for each indicator and control variable. This lag structure was applied consistently across both panel-level and country-specific estimations, ensuring methodological coherence, preserving degrees of freedom, and maintaining comparability of results across specifications.

Table 2 presents the PMG-ARDL estimates of the relationship between the EDB index and both FDI and domestic investment, capturing both short-run and long-run effects. The long-term estimates reveal a modest yet statistically significant negative coefficient for the relationship between the EDB index and FDI (–0.024), a finding that runs counter to conventional expectations [18,19]. Comparable paradoxical or null effects have been documented in prior empirical studies, particularly in contexts where comprehensive regulatory overhauls have introduced a phase of transitional uncertainty and elevated compliance costs that temporarily outweigh the anticipated benefits of reform [39,62]. In emerging and reforming economies, abrupt changes to the regulatory framework can disrupt established business networks, compel firms to adapt to new administrative, legal, and reporting requirements, and thereby raise the effective cost of entry or operation in the short run. In addition, improvements in the aggregate business climate are sometimes accompanied by the withdrawal or reduction of targeted FDI incentives, such as tax holidays, preferential financing, or sector-specific subsidies, that had been instrumental in attracting foreign capital in the pre-reform period [40]. This substitution of general business facilitation for specific inducements can diminish immediate inflows, even when the underlying regulatory environment is objectively more conducive to investment. Furthermore, the aggregate EDB index may obscure heterogeneity in the effects of its subcomponents; as our disaggregated results show, foreign investors tend to respond positively to improvements in enforcing contracts, trading across borders, and resolving insolvency, but may perceive stricter requirements in starting a business, registering property, or paying taxes as near-term impediments [27,83]. From a policy standpoint, these results suggest that regulatory reforms, while necessary, must be carefully sequenced and embedded within a broader institutional strengthening agenda in order to mitigate their short-term deterrent effects. Reform packages should be introduced gradually to allow investors adequate time to adjust, with transitional arrangements, such as grandfathering clauses, phased implementation schedules, or temporary compliance relief, designed to minimise adjustment costs. The removal of targeted FDI incentives should be timed in accordance with demonstrable improvements in institutional capacity and the consolidation of the new regulatory framework, rather than implemented abruptly. Policymakers should also prioritise those dimensions of the business environment that exhibit the strongest and most consistent positive associations with FDI inflows, particularly contract enforcement, cross-border trade facilitation, and predictable insolvency procedures, while ensuring that tax and property registration reforms do not impose disproportionate burdens on new entrants. Crucially, enhancements to the EDB framework should proceed in tandem with sustained improvements in governance effectiveness, regulatory quality, and control of corruption [22], as these institutional factors can significantly reduce the duration of uncertainty and facilitate the translation of business-environment reforms into durable capital inflows.

**Table 2:** The impact of aggregate EDB scores on FDI and domestic investment.

EDB-FDI LINK					EDB-DI LINK			
Variables	Coefficients	Std. Err.	z	P > z	Coefficients	Std. Err.	z	P > z
Long Run								
EDB	−0.024*	0.010	−2.44	0.015	0.497*	0.174	2.86	0.004
GDP	−0.021	0.036	−0.58	0.560	2.240*	0.465	4.81	0.000
TRA	0.067*	0.011	6.15	0.000	0.401*	0.122	3.28	0.001
NRT	0.159*	0.037	4.3	0.000	−0.241*	0.076	−3.18	0.001
XC	0.001	0.001	−0.91	0.360	0.001	0.001	0.99	0.322
Short Run								
ECM	−0.484*	0.165	−2.94	0.003	−0.134*	0.039	−3.42	0.001
dEDB	−0.108	0.106	−1.02	0.306	−0.139	0.238	−0.59	0.558
dGDP	0.126	0.095	1.33	0.182	−0.085	0.126	−0.68	0.498
dTRA	−0.049	0.033	−1.51	0.131	−0.079	0.108	−0.73	0.463
dNRT	−0.001	0.039	−0.01	0.989	−0.089	0.264	−0.34	0.737
dXC	0.072	0.048	1.51	0.130	−0.173	0.190	−0.91	0.363
_cons	−0.384	0.324	−1.19	0.236	−3.193*	1.259	−2.54	0.011

Note: *** p < 0.01, ** p < 0.05, * p < 0.1. Author’s calculations.

In addition, trade openness and natural resources exhibit positive long-term effects on FDI, with coefficients of 0.067 and 0.159, respectively. This indicates that economies with higher levels of trade integration and abundant natural resources are more likely to attract FDI. Open trade policies facilitate easier access to global markets, while resource-rich countries offer lucrative opportunities in extraction and related sectors [112,113]. These findings echo those of Albahouth and Tahir [114], who found that trade openness significantly enhances FDI inflows in ASEAN region over the long term. Similarly, Mayoshi and Epuran [115] observed that trade openness has a positive and statistically significant impact on FDI in emerging Asian economies.

Conversely, the EDB index’s long-term effect on domestic investment is markedly positive, with a significant coefficient of 0.497, underscoring that a refined business environment characterized by streamlined regulations and reduced compliance complexities catalyzes domestic investment. Such an environment lowers entry barriers and fosters predictability, encouraging local businesses to invest and grow. This positive correlation between the EDB index and domestic investment is supported by Anggraini and Inaba [73] assertion that a comprehensive EDB score is instrumental in attracting inward FDI. While their focus is on FDI, the implication is clear: an enhanced business climate also bodes well for domestic investment. Kalai et al. [116] further reinforce this notion, demonstrating that economic growth is boosted when certain thresholds are exceeded, suggesting that improvements in the EDB index, which typically lead to increased trade and investment, favour domestic investment.

Additionally, market size and TRA exhibit positive coefficients (2.240 and 0.401), respectively, aligning with the expectation that a robust GDP and trade integration are catalysts for domestic investment [117–121]. A large market size signifies a strong economy with a vast consumer base, enticing businesses to invest to meet increasing demands. This economic vigor fosters investor confidence and suggests an efficient allocation of resources, including investment capital. Furthermore, trade openness facilitates local firms’ access to international markets, encouraging investments in export-oriented sectors. This dynamic creates a conducive environment for domestic ventures, where a thriving market size and integrated trade systems act as dual engines propelling domestic investment forward. Conversely, the negative coefficient for NRT (−0.241) hints at the “resource curse,” where an overabundance of natural resources might inadvertently stifle investment in diverse economic sectors. This phenomenon can lead to Dutch disease, where resource-driven revenue skews the economy, impedes diversification, and crowd out investment in non-resource industries [122].

In the short term, the Error Correction Terms (ECM) for FDI (–0.484) and DI (–0.134) are both negative and statistically significant at the 5% level. These values indicate that around 48% of deviations in FDI and 13% in DI from their respective long-run equilibria are corrected within a year. The faster adjustment in FDI suggests that foreign investment flows respond more quickly to disequilibria, while DI adjustments are slower, allowing short-term deviations to persist. The negative coefficients confirm the expected mean-reverting behaviour, where market and policy mechanisms act to restore equilibrium after shocks.

Beyond the speed of adjustment, the estimated short-run coefficients show that neither the EDB index nor the control variables have statistically significant effects on FDI or DI. This lack of immediate impact suggests that improvements in the ease of doing business and related macroeconomic factors tend to influence investment decisions gradually, as policy changes and regulatory reforms require time to take hold. In this context, the short-run dynamics reinforce the idea that the main channel through which EDB reforms affect investment is the long-term adjustment process rather than immediate responses.

These findings partially support Hypothesis 1. While the ease of doing business index has a significant long-term positive relationship with domestic investment, its long-term association with FDI is unexpectedly negative, indicating that the hypothesized uniformly positive effect applies to domestic investment but not to foreign investment in the BRICS+ context.

#### 5.3.2 The impact of ease of doing business indicators on FDI and domestic investment.

Table 3 delineates the outcomes of EDB indicators on both FDI and domestic investment. In the context of FDI, the long-term effects reveal that the indicators for Starting a Business (SB), Registering Property (RP), and Paying Taxes (PT) register negative and statistically significant coefficients, implying that enhancements in these areas tend to deter FDI inflows over an extended period. The apparent paradoxical result could be attributed to how foreign investors perceive stricter regulations in these areas. Some investors may see these regulations as potential obstacles or added expenses, which might eclipse the advantages of streamlined procedures. On the other hand, better performance in facilitating cross-border trade, enforcing contracts, and resolving insolvency have positive long-term effects on FDI. These findings are congruent with the expectation that foreign investors value efficient trade processes and reliable contract enforcement mechanisms. Additionally, a larger market size (GDP) attracts more FDI in the long run, suggesting foreign investors are drawn to growing economies. Interestingly, resolving insolvency and market size have negative and significant coefficients in the short run, which contradicts their long-run effects. This discrepancy could be attributed to the initial adjustment costs or disruptions associated with implementing reforms in insolvency procedures or temporary fluctuations in economic growth.

**Table 3:** The impact of EDB indicators on FDI and domestic investment.

PMG	EDB Indicators-FDI Nexus			EDB Indicators-DI Nexus				
Variables	Coefficients	Std. Err.	z	P > z	Coefficients	Std. Err.	z	P > z
Long-Run								
SB	−0.107*	0.030	−3.57	0.000	−0.967*	0.338	−2.86	0.004
DCP	−0.020	0.024	−0.83	0.406	0.214	0.226	0.95	0.344
GEL	−0.006	0.034	−0.19	0.853	−0.531***	0.289	−1.83	0.067
RP	−0.131*	0.046	−2.82	0.005	1.389*	0.519	2.68	0.007
GC	0.022	0.027	0.82	0.412	−0.027	0.209	−0.13	0.897
PIM	−0.028	0.044	−0.63	0.53	0.661***	0.383	1.73	0.084
PT	−0.100**	0.049	−2.03	0.042	−1.039**	0.459	−2.26	0.024
TAB	0.057**	0.029	2.01	0.044	0.561**	0.230	2.43	0.015
EC	0.090**	0.039	2.33	0.02	0.283	0.307	0.92	0.357
RI	0.239*	0.078	3.06	0.002	−0.020	0.578	−0.03	0.973
GDP	0.178*	0.053	3.38	0.001	0.322	0.412	0.78	0.434
TRA	−0.017**	0.020	−0.86	0.391	0.058	0.160	0.36	0.719
NRT	−0.062	0.042	−1.46	0.144	−0.280	0.328	−0.85	0.394
XC	0.001	0.001	0.25	0.800	0.000	0.000	−1.00	0.319
Short-Run								
ECM	−0.592*	0.072	−8.19	0.000	−0.229*	0.069	−3.32	0.001
dSB	0.022	0.021	1.03	0.303	0.037	0.066	0.56	0.572
dDCP	−0.001	0.018	−0.08	0.938	0.028	0.054	0.52	0.604
dCP	−0.010	0.023	−0.42	0.674	−0.011	0.070	−0.15	0.877
dGE	0.011	0.038	0.29	0.771	−0.215***	0.119	−1.8	0.072
dRP	−0.016	0.017	−0.98	0.33	−0.073	0.051	−1.43	0.151
dPIM	0.019	0.032	0.6	0.546	−0.107	0.099	−1.08	0.282
dPT	0.040	0.031	1.31	0.189	0.297*	0.095	3.13	0.002
dTAB	−0.003	0.023	−0.13	0.893	−0.025	0.070	−0.37	0.714
dEC	−0.032	0.034	−0.94	0.345	−0.062	0.104	−0.59	0.553
dRI	−0.084***	0.048	−1.73	0.084	0.036	0.149	0.24	0.809
dGDP	−0.048***	0.028	−1.71	0.087	0.010	0.083	0.12	0.908
dTRA	0.001	0.017	0.07	0.942	0.055	0.053	1.04	0.297
dNRT	0.017	0.024	0.72	0.474	−0.037	0.078	−0.48	0.634
dXC	0.000	0.000	−0.01	0.994	0.000	0.000	−0.69	0.488
_cons	5.506**	2.477	2.22	0.026	4.690	7.756	0.6	0.545

Note: *** p < 0.01, ** p < 0.05, * p < 0.1. Author’s calculations.

Existing research also provides a mixed picture of the relationship between EDB indicators and FDI. While some studies, such as those by Hassan and Basit [62] and Bardakas et al. [39], find negative impacts of specific indicators like registering property and paying taxes, others, like Njuguna and Nnadozie [19] and Corcoran and Gillanders [27], highlighted the positive role of a conducive business environment, particularly efficient trade processes. Notably, Hassan and Basit [62] also suggested that strong contract enforcement, facilitated by a favorable business environment, attracts FDI. Morris and Aziz [123] identified registering property and trading across borders as relevant factors for FDI. These findings underscore the complexity of the relationship between the business environment and FDI, where different indicators can have varying effects, influenced by specific contexts and regions.

Similar to FDI, the long-term effects on domestic investment paint a surprising picture. Improvements in starting a business, obtaining electricity, registering property, and paying taxes record negative coefficients, implying that, in the long run, streamlining these processes might discourage domestic investment. Like foreign investors, domestic actors might perceive stricter regulations as potential hurdles or added costs, even if they simplify procedures. However, there are positive long-term influences as well. Stronger protections for minority investors and efficient cross-border trade procedures attract more domestic investment, suggesting that domestic investors value a legal framework that safeguards their rights and a business environment that facilitates trade.

In the short term, the Error Correction Terms (ECM) for FDI (–0.592) and DI (–0.229) are both negative and statistically significant at the 1% level. These values imply that approximately 59% of deviations in FDI and 23% in DI from their respective long-run equilibria are corrected within a year. The faster speed of adjustment for FDI suggests greater responsiveness of foreign capital flows to disequilibria, while DI corrections are more gradual. The negative coefficients confirm the expected mean-reverting behaviour, indicating that market and policy mechanisms work to restore equilibrium when investment levels deviate from their long-run path.

In terms of short-run effects, most EDB indicators and control variables are insignificant for both FDI and DI, suggesting that their impact emerges mainly over the long term. Notable exceptions include getting electricity, which exerts a negative short-run effect on DI (–0.215), possibly due to transitional disruptions or infrastructure upgrade costs, and paying taxes, which has a positive short-run effect on DI (0.297), indicating an initially favourable investor response to tax reforms before longer-term implications become apparent. Short-run effects on FDI are negligible, supporting the view that EDB reforms influence foreign investment primarily through long-run channels..

### 5.3 Country-specific analysis

The results from Table 4 reveal diverse effects of the aggregate EDB index on both FDI and domestic investment across the different countries. With the FDI model, China and Iran stand out with positive and statistically significant EDB coefficients, signaling that an enhancement in the business environment is likely to bolster FDI inflows. Conversely, Ethiopia presents a counterintuitive scenario where an improved EDB index correlates with reduced FDI inflows, as indicated by a negative coefficient, suggesting that other factors may overshadow the presumed benefits of a favorable business climate. Meanwhile, Brazil, Egypt, India, Russia, South Africa, and the United Arab Emirates show statistically insignificant EDB coefficients, hinting that the overall business environment may not be pivotal in attracting FDI in these economies. Regarding domestic investment, Brazil, China, and Iran reveal negative EDB coefficients, contrary to theoretical expectations that posit an improvement in the business environment should stimulate investment. This suggests that such enhancements might dampen domestic investment levels initially due to potential adjustment costs or other transitional challenges. The remaining countries show insignificant effects of the EDB index on domestic investment.

**Table 4:** Country-specific analysis of the impact of the EDB index on investment.

Country	ECM	dEDB	dGDP	dTRA	dNRT	dXC	Key Insights
FDI Model							
Brazil	−0.09	0.152	0.155**	0.054	0.028	0.171	GDP influences FDI; ECM is not significant
China	−1.246*	0.045*	0.569	0.037	−0.062	0.221	Strong ECM correction; EDB positive effect
Egypt	−0.517*	0.043	0.833*	0.007	−0.036	0.048	GDP is the main FDI driver
Ethiopia	−0.168	−0.659*	0.218	−0.062	−0.16	0.332	EDB is negatively associated with FDI
India	−0.360***	0.012	−0.082	−0.059	0.259	0.046	Moderate ECM; coefficients mostly weak
Iran	0.200*	0.075*	−0.010***	−0.023**	0.018**	0.000	Positive EDB impact; GDP has a negative sign
Russia	−0.733*	−0.056	0.071	−0.277**	0.051	−0.035	Strong ECM; trade openness negatively impacts
South Africa	−1.198*	0.074	−0.07	−0.03	−0.079	−0.132	ECM is strong; no major short-run drivers
UAE	−0.241***	−0.661*	−0.036	−0.092*	−0.023	0.000	EDB negatively associated; ECM significant
DI Model							
Brazil	−0.208*	−0.581*	0.033	0.135**	−0.01	0.033	EDB negatively associated; trade matters
China	0.036	−0.243*	0.249	−0.254*	0.224	−1.492	EDB negative; trade openness hurts DI
Egypt	−0.251*	−0.141	0.389**	0.038	0.158	0.121	GDP moderately influences DI
Ethiopia	−0.191**	−1.289	−0.828	−0.116	0.846	0.481	Negative GDP link; EDB effect inconclusive
India	−0.067	−0.01	−0.133	0.409*	−2.005*	−0.629*	Trade openness and NRT shape DI
Iran	−0.246**	1.450*	−0.326***	−0.768**	0.285	0.000	Strong EDB positive effect
Russia	−0.085**	−0.159	0.291*	−0.188	0.039	0.022	GDP supports DI; weak trade link
South Africa	−0.236*	−0.155	−0.256*	−0.008	0.101	−0.095	Negative GDP link; ECM mild
UAE	0.039**	−0.127	−0.189*	0.040	−0.438*	0.000	EDB and NRT are negatively associated with DI

Note: *** p < 0.01, ** p < 0.05, * p < 0.1. Author’s calculations.

**Fig 3:**
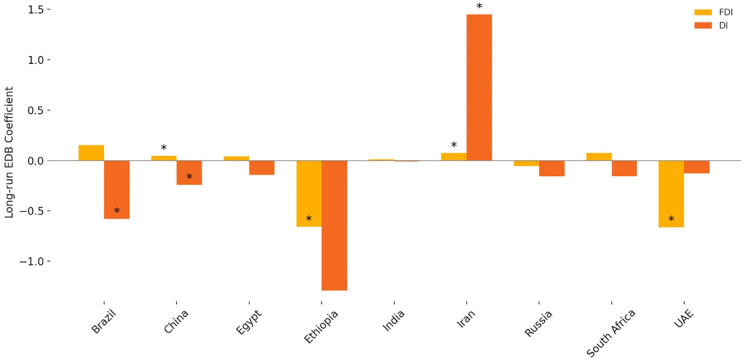
Country-specific long-run effects of EDBon FDI and DI in BRICS+ countries. NB: * indicates statistically significant coefficients (p < 0.1). Source: Authors’ estimates using the PMG-ARDL model.

Further analysis of other determinants unveils that GDP growth, a proxy for market size, has a positive and statistically significant effect on Brazil’s FDI, Egypt’s FDI, and Russia’s DI, aligning with the conventional wisdom that economic expansion fosters higher investment levels [124]. Similarly, trade openness is positively correlated with increased investment levels in Brazil (DI), India (DI), and Iran (FDI), underscoring the role of trade integration in investment dynamics. Iran’s natural resource endowments also emerge as a significant attractor of FDI. The overarching narrative from these results is substantial heterogeneity in how the EDB index and other determinants impact FDI and domestic investment across different countries. Fig 3 visually summarises these results, which compares the long-run EDB coefficients for FDI and DI across countries. Significant levels are indicated to highlight where reforms appear most impactful.

### 5.4 Mediation analysis

Table 5 outlines the indirect influences of the EDB index on FDI and domestic investment, mediated by regulatory quality, government effectiveness, and control of corruption. The Sobel test confirms these effects’ significance, quantifying two ratios: RIT (ratio of indirect to total effect) and RID (ratio of indirect to direct effect). Regulatory quality is a significant conduit for EDB’s positive impact on FDI, with a notable RIT value of 2.183, indicating that over twice the total EDB effect on FDI operates through this pathway. The RID value of 0.686 means the mediated effect surpasses the direct impact by nearly 70%. Similarly, regulatory quality significantly mediates EDB’s effect on DI, with an RIT of 4.376, suggesting that regulatory improvements channel over four times the total EDB effect on DI. The RID value of 1.296 indicates that this indirect effect is around 30% larger than the direct impact.

**Table 5:** Mediation analysis.

	Indirect Effect	Significance testing of indirect effect (unstandardized)	
Model	Sobel Estimates	RIT = (Indirect effect/ Total effect)	RID = (Indirect effect/ Direct effect)
Regulatory Quality as a Mediator			
RQ→EODB→FDI	0.057 *	(0.057/ 0.026) = 2.183	(0.057/ 0.084) = 0.686
RQ→EODB→DI	-0.318 *	(0.318/ 0.073) = 4.376	(0.318/ 0.245) = 1.296
Government Effectiveness as a Mediator			
GE→EODB→FDI	0.044*	(0.044/ 0.026) = 1.667	(0.044/ 0.070) = 0.625
GE→EODB→DI	0.092 *	(0.092/ 0.073) = 1.267	(0.092/ 0.165) = 0.559
Corruption Control as a Mediator			
CC→EODB→FDI	0.022*	(0.022/ 0.026) = 0.825	(0.022/ 0.048) = 0.452
CC→EODB→DI	-0.080***	(0.080/ 0.073) = 1.097	(0.080/ 0.007) = 11.333

Note: *** p < 0.01, ** p < 0.05, * p < 0.1. Author’s calculations.

Government Effectiveness also significantly mediates EDB’s positive influence on FDI, with an RIT of 1.667, implying that about 167% of EDB’s total effect on FDI is through effective governance. The RID value of 0.625 shows that this mediated effect is around 60% as large as the direct impact. This pattern also holds for DI, where government effectiveness mediates around 127% of EDB’s total effect on DI (RIT value), and the mediated effect is about 56% as large as the direct impact (RID value).

Control of Corruption reveals a significant positive mediation of EDB’s effect on FDI, with an RIT value of 0.825, indicating that controlling corruption accounts for about 82% of EDB’s total impact on FDI. The RID value of 0.452 shows that this mediated effect is nearly half as large as the direct impact. For DI, while the mediation through corruption control is weakly significant, it carries substantial weight: an RIT value of 1.097 suggests that over 100% of EDB’s total effect on DI is through this channel. An astonishing RID value of 11.333 indicates that the mediated effect is more than eleven times larger than the direct impact, signifying that controlling corruption plays a particularly crucial role in domestic investment, potentially outweighing the direct effects of EDB improvements.

Impliedly, the results provide strong empirical support for Hypothesis 2, confirming that regulatory quality significantly mediates the positive relationship between the EDB index and both FDI and domestic investment, with mediated effects exceeding the magnitude of direct effects in both cases. Similarly, the findings support Hypothesis 3, as government effectiveness emerges as a significant mediating channel through which EDB improvements enhance both forms of investment. Finally, the evidence also supports Hypothesis 4, with control of corruption mediating the EDB-investment relationship for FDI and, though more weakly significant for domestic investment, exerting an indirect influence of exceptional magnitude, particularly in the domestic investment context.

### 5.5 Moderating analysis

Table 6 displays the moderating effects of financial development, economic freedom, and economic stability (inflation) on the relationship between the EDB, FDI and domestic investment. Financial development amplifies the positive influence of the EDB on FDI and domestic investment in the long term, as evidenced by the significant positive coefficients found in the interaction terms, suggesting that a robust financial system enhances investment by improving credit access and resource allocation. In contrast, higher levels of economic freedom unexpectedly attenuate the positive effect of the EDB index on both FDI and domestic investment. The negative coefficients on the interaction terms suggest that in the BRICS+ context, certain dimensions of economic freedom, such as rapid deregulation, shifts in trade or capital account policies, or changes in property rights enforcement, may create transitional uncertainties that outweigh the immediate benefits of a more liberalized environment. For instance, the relaxation of regulatory controls without parallel strengthening of institutional oversight can increase exposure to market volatility, policy reversals, or uneven enforcement, conditions that may deter both foreign and domestic investors. Additionally, liberalization measures in labor markets or foreign ownership rules, if implemented abruptly, may require substantial adaptation from firms, thereby dampening the short-term responsiveness of investment to improvements in the ease of doing business. Moreover, rising inflation rates are shown to weaken the positive impact of EDB on FDI, aligning with expectations that inflation creates uncertainty and diminishes investment attractiveness. However, inflation does not significantly moderate the relationship between EDB and DI, indicating that domestic investment may be less sensitive to inflationary pressures in these economies.

**Table 6:** Moderating analysis.

EDB-FDI LINK					EDB-DI LINK			
Variables	Coefficients	Std. Err.	z	P > z	Coefficients	Std. Err.	z	P > z
Long Run								
EDB	−0.0287	0.0838	−0.34	0.732	0.3792*	0.0608	6.2300	0.0000
GDP	0.4010*	0.0747	5.37	0.000	0.7183*	0.0781	9.1900	0.0000
TRA	−0.1149*	0.0148	−7.76	0.000	0.1817*	0.0278	6.5300	0.0000
NRT	0.5676*	0.0884	6.42	0.000	−0.5004*	0.0483	−10.360	0.0000
XC	0.0787*	0.0182	4.31	0.000	0.0000	0.0001	0.2900	0.7720
EDBFID	0.0014*	0.0004	3.34	0.001	0.0037*	0.0004	10.4200	0.0000
EDBEFI	−0.0037*	0.0014	−2.75	0.006	−0.0041*	0.0009	−4.7600	0.0000
EDBINF	−0.0064*	0.0007	−9.35	0.000	0.0002	0.0003	0.7500	0.4540
Short Run								
ECM	−0.2303**	0.1089	−2.11	0.035	−0.3603**	0.1582	−2.28	0.0230
dEDB	−0.0848	0.0964	−0.88	0.379	−0.3348	0.3639	−0.92	0.3580
dGDP	0.1109	0.0861	1.29	0.198	−0.1379	0.1408	−0.98	0.3270
dTRA	−0.0427	0.0446	−0.96	0.338	−0.1343	0.1068	−1.26	0.2080
dNRT	−0.0903	0.0611	−1.48	0.139	0.2258	0.2968	0.76	0.447
dXC	0.0886	0.2032	0.44	0.663	−0.4309**	0.1980	−2.18	0.03
dEDBFID	0.0019	0.0015	1.24	0.214	0.0090	0.0100	0.9	0.369
dEDBEFI	−0.0008	0.0012	−0.67	0.504	−0.0002	0.0024	−0.1	0.918
dEDBES	0.0007	0.0008	0.78	0.434	0.0002	0.0009	0.29	0.775
_cons	2.8323*	1.4485	1.96	0.051	−1.2347	2.1046	−0.59	0.557

Note: *** p < 0.01, ** p < 0.05, * p < 0.1. EDBFID is the interaction term between EDB and Financial development, EDBEFI is the interaction term between EDB and Economic freedom, and EDBES is the interaction term between EDB and economic stability—Author’s calculations.

Jointly, these findings support Hypothesis 5, confirming that financial development significantly strengthens the positive relationship between the EDB index and both FDI and domestic investment, consistent with the role of well-functioning financial systems in translating regulatory improvements into tangible investment gains. The results, however, do not support Hypothesis 6 in its expected form as higher economic freedom unexpectedly attenuates the positive effect of the EDB index on investment, suggesting that certain facets of economic liberalization may introduce frictions or uncertainties for investors in the BRICS+ context. Finally, the evidence partially supports Hypothesis 7, as economic stability, proxied by inflation, weakens the EDB–FDI relationship as anticipated, but shows no statistically significant moderating effect on domestic investment, indicating a differentiated sensitivity between foreign and domestic capital to macroeconomic volatility.

## 6. Conclusion and policy recommendations

Within the intricate tapestry of global economic dynamics, the BRICS consortium, augmented by the recent inclusion of nations such as Egypt, Ethiopia, Iran, and the United Arab Emirates, has emerged as a formidable collective influencing investment trajectory. These nations, accounting for a substantial fraction of global GDP and demographic heft, have experienced an impressive rise in their economic clout. Their capacity to attract and sustain FDI and stimulate domestic investment hinges on a confluence of factors, including the ease of doing business, prevailing macroeconomic conditions, and the strength of institutional frameworks. This study examined the effects of EDB on investment flows (both FDI and domestic investment) in BRICS economies with three core objectives. First, it assessed the direct influence of EDB on investment. Second, it explored the mediating role of institutional quality, focusing on regulatory quality, government effectiveness, and corruption control. Third, it investigated how financial development, economic freedom, and inflation moderate the EDB-investment relationship. By employing a combination of PMG-ARDL and Structural Equation Modeling (SEM) on panel data from 2004 to 2020, the study offers an in-depth, mechanism-based understanding of how regulatory and institutional environments shape investment behavior.

This work makes several distinct contributions to the literature on investment and institutional reform in emerging economies. It is among the few to examine the direct, mediated, and moderated effects of EDB on investment flows using an integrated PMG-ARDL and SEM framework. By combining panel dynamics with causal pathway analysis, the study provides a more holistic and nuanced view of how business environment reforms interact with institutional and macroeconomic factors. It extends the analysis beyond FDI to include domestic investment, offering a fuller perspective on capital formation. In addition, it disaggregates the EDB index to examine the impact of individual business environment components and reveals substantial country-level heterogeneity. The inclusion of newly admitted BRICS+ countries, Egypt, Ethiopia, Iran, and the UAE, broadens the empirical scope, enhancing both the regional relevance and the global policy applicability of the findings. Based on the results, the evidence supports Hypotheses 1, 2, 3, 4, 5, and 7, while Hypothesis 6 is not supported, as economic freedom is found to moderate the EDB-investment relationship negatively.

The empirical findings offer several pivotal insights. The EDB index exhibits a dualistic effect. While it promotes domestic investment, its long-term impact on FDI is negative. This result may be explained by short-run adjustment costs associated with regulatory reform or by the withdrawal of investment incentives that had previously compensated for institutional weaknesses. The disaggregated analysis further reveals that some EDB indicators, while essential for improving operational efficiency, may be perceived by foreign investors as burdensome or restrictive, thereby inadvertently reducing FDI attractiveness. In contrast, market size and trade openness have positive effects on domestic investment, while natural resource abundance is negatively associated with investment, indicating potential resource curse dynamics. Institutional quality variables such as regulatory quality, governance effectiveness, and corruption control emerge as potent mediators in the EDB-investment relationship, with indirect effects outweighing the direct impact of EDB reforms. Lastly, the moderation analysis revealed that financial development bolsters the positive link between ease of doing business and investment. In contrast, economic freedom dampened this effect, with inflation found to affect FDI adversely and having an insignificant impact on domestic investment.

Based on these findings, several important policy considerations emerge. First, improvements in the ease of doing business must be pursued in tandem with institutional strengthening. Regulatory reforms that are not supported by improvements in legal enforcement, bureaucratic capacity, and anti-corruption measures may be seen as symbolic or unsustainable, thereby reducing investor confidence. The strong mediating role of institutional quality in this study underscores the need for integrated reform strategies that address both procedural and systemic constraints.

Second, the observed negative long-term association between the EDB index and FDI suggests that regulatory transitions must be carefully managed. Governments should avoid prematurely phasing out FDI-targeted incentives before the long-run benefits of business environment reforms are fully realized. Countries undertaking major reforms must account for short-term investor uncertainty and design transitional policies that maintain investor confidence. Phased implementation, coupled with proactive investor engagement and policy transparency, can help mitigate the adjustment costs associated with reform.

Third, the heterogeneity of results across countries further reinforces the importance of context-sensitive reforms. China and Iran, where EDB improvements are positively associated with FDI, should sustain regulatory streamlining while introducing targeted sectoral incentives to consolidate these gains. Ethiopia, where EDB reforms currently correlate with reduced FDI inflows, must first address complementary constraints such as political stability, infrastructure gaps, and policy predictability before relying on regulatory reform as the main driver of foreign investment. Brazil and Egypt can leverage their large domestic markets by improving credit access and trade logistics to strengthen domestic investment, while also addressing the insignificant EDB–FDI link through focused investor facilitation programs. India would benefit from deepening trade openness to support domestic investment and pairing EDB reforms with infrastructure improvements. Russia and South Africa, where trade openness and GDP are important drivers, could prioritize export infrastructure and policies that reduce investor uncertainty in sectors beyond natural resources. For the UAE, diversifying investment drivers beyond hydrocarbons and ensuring that EDB reforms yield benefits for both foreign and domestic investors would help strengthen resilience.

Fourth, the positive moderating effect of financial development on the EDB-investment relationship underscores the importance of a vibrant financial sector. Improving access to credit, strengthening banking regulations, and expanding financial inclusion are all critical to unlocking the full potential of EDB reforms. Without robust financial intermediation, even the most business-friendly regulations may fail to translate into tangible investment outcomes. Policymakers must, therefore, view financial development not as a separate agenda but as a vital enabler of business environment reforms.

Lastly, the negative influence of inflation and the complex effects of economic freedom on investment highlight the need for macroeconomic stability and well-calibrated liberalization. While economic freedom is generally beneficial, excessive or unstructured deregulation may create uncertainty or weaken institutional safeguards. Sound monetary policy and inflation control are essential to reduce investor risk perceptions. Moreover, regional cooperation within BRICS+, through harmonized investment regulations and policy frameworks, can enhance the predictability of the cross-border investment environment and strengthen the bloc’s collective appeal to global investors.

While this study provides new insights into the institutional and macroeconomic mechanisms linking EDB reforms to investment in BRICS+ economies, two avenues warrant further investigation. First, with the discontinuation of the World Bank’s EDB index in 2021, subsequent studies could employ alternative composite indicators of business environment quality to examine whether the relationships observed here persist in the post-2020 context. Second, sector-specific analyses of FDI and domestic investment responses to regulatory reforms could provide a more granular understanding of which industries benefit most from particular dimensions of the business environment, thereby enabling more targeted policy interventions.

## Supporting information

S1 Table Definition of variables. (DOCX)

S2 Table List of BRICS countries. (DOCX)

S3 Table Descriptive statistics. (DOCX)

S4 Table Pearson correlation matrix analysis. (DOCX)

S5 Table Cross-sectional dependence test results. (DOCX)

S6 Table Variance Inflation Factor (VIF) and slope heterogeneity tests. (DOCX)

S7 Table Panel unit root tests. (DOCX)

S8 Table Westerlund ECM panel cointegration tests. (DOCX)

S1 File The means of the ease of doing business (EDB) index and its indicators. (DOCX)
