# Supplementary material for: Investment attractiveness in BRICS+ economies: Evaluating business environment reforms, institutional quality, and macroeconomic factors
Source: PLoS One. 2025 Oct 16;20(10):e0334043. doi: 10.1371/journal.pone.0334043 (PMC12530542; doi:10.1371/journal.pone.0334043)
Supplement: S1 Table — (DOCX) [file pone.0334043.s001.docx]

# **S1 Table. Definition of Variables**

S1 Table. Definition of Variables

| Indicator | Description |
| --- | --- |
| Ease of Doing Business | The Ease of Doing Business Index is calculated as the straightforward average of scores assigned to various components, which include starting a business, obtaining construction permits, accessing electricity, property registration, credit acquisition, minority investor protection, cross-border trade, contract enforcement, insolvency resolution, and tax payment process. |
| Starting a Business | Procedures, time, cost, and minimum capital to open a new business |
| Dealing with Construction Permits | Procedures, time, and cost to build a warehouse |
| Getting Electricity | Procedures, time, and cost required for a business to obtain a permanent electricity connection |
| Registering Property | Procedures, time, and cost to register commercial real estate |
| Getting Credit | Movable collateral laws and credit information systems |
| Protecting Minority Investors | Minority shareholders’ rights in related-party transactions and in corporate governance |
| Paying Taxes | Taxes and contributions payable by businesses after accounting for allowable deductions and exemptions |
| Trading Across Borders | Time and cost to export the product of comparative advantage and import auto parts |
| Enforcing Contracts | Time and cost to resolve a commercial dispute |
| Resolving Insolvency | Time, cost, and outcome of insolvency proceedings |
| Market Size | Market size’ denoted by GDP, refers to the total value of all goods and services produced within a country’s economy over a specific period, which indicates the overall market capacity of the country. It reflects the economic strength and potential for businesses operating within that market |
| Regulatory Quality | Reflects perceptions of the ability of the government to formulate and implement sound policies and regulations that permit and promote private sector development |
| Governance Effectiveness | Captures perceptions of the quality of public services, the quality of the civil service and the degree of its independence from political pressures, the quality of policy formulation and implementation, and the credibility of the government’s commitment to such policies. |
| Control of Corruption | Measures the extent to which public power is exercised for private gain, including both petty and grand forms of corruption, as well as “capture” of the state by elites and private interests |
| Financial Development | Measured by the domestic credit provided by the financial sector as a percentage of GDP. It indicates the financial services banks and other financial institutions provide to businesses and individuals. |
| Economic Freedom Index | This index scores countries based on factors such as rule of law, government size, regulatory efficiency, and open markets, and it measures the level of economic freedom in a country. Economic freedom refers to the degree of government intervention in the economy and the freedom of individuals and businesses to participate in economic activities. |
| Economic Stability | This is often gauged by the annual percentage change in consumer prices, reflecting inflation. Low and stable inflation is typically a sign of economic stability. |
|  |  |
