# Supplementary material for: Investment attractiveness in BRICS+ economies: Evaluating business environment reforms, institutional quality, and macroeconomic factors
Source: PLoS One. 2025 Oct 16;20(10):e0334043. doi: 10.1371/journal.pone.0334043 (PMC12530542; doi:10.1371/journal.pone.0334043)
Supplement: S2 Table — (DOCX) [file pone.0334043.s002.docx]

# S2 Table. List of BRICS Countries

S2 Table. List of BRICS Countries

| Founding Members (2006) | Newly Joined Members (2024) |
| --- | --- |
| Brazil | Iran |
| Russia | Ethiopia |
| India | Egypt |
| China | United Arab Emirates |
| South Africa |  |

*South Africa joined the group in 2010
