# Supplementary material for: Investment attractiveness in BRICS+ economies: Evaluating business environment reforms, institutional quality, and macroeconomic factors
Source: PLoS One. 2025 Oct 16;20(10):e0334043. doi: 10.1371/journal.pone.0334043 (PMC12530542; doi:10.1371/journal.pone.0334043)
Supplement: S3 Table — (DOCX) [file pone.0334043.s003.docx]

**S3 Table. Descriptive Statistics**

S3 Table presents a statistical overview of key economic indicators for nine countries, highlighting their economic and institutional diversity. Foreign Direct Investment (FDI) varies widely, with Iran at the lower end (mean of 0.7548) and UAE at the higher end (mean of 3.447), indicating different levels of foreign investment. Brazil, Egypt, Ethiopia, and UAE show a right-skewed FDI distribution, suggesting a tendency towards higher values. In contrast, Brazil’s negative skewness points to a clustering of values below the mean. India and Iran have high kurtosis in FDI, suggesting a greater chance of outliers. Domestic Investment (DI) also shows significant variation, with China having the highest mean (43.598) and Egypt the lowest (17.517), reflecting different domestic investment levels. The Ease of Doing Business index ranges notably, with UAE at the top (mean of 73.238) and Ethiopia at the bottom (mean of 45.973). Negative skewness in Egypt and Ethiopia indicates most observations fall below the mean. GDP growth rates vary, with China and Ethiopia leading, while the UAE’s negative growth rate reflects its dependence on non-renewable resources. India’s negative skewness in GDP growth suggests lower rates are more common. Trade values differ significantly, with UAE’s high mean (151.227) highlighting its trade hub status. Natural Resource metrics show Egypt and China in the lead, with South Africa’s positive skewness indicating a distribution skewed towards higher values. Exchange Rate averages cover a broad spectrum, with Iran’s high mean (20631.88) possibly due to economic factors like hyperinflation or currency depreciation. Regulatory Quality (RQ), Government Effectiveness (GE), and Control of Corruption (CC) show varied means across countries, with UAE ranking high, reflecting robust governance structures. Most countries have negative means for these governance indicators, suggesting room for improvement. The descriptive statistics reveal significant differences in economic performance and governance quality among the countries studied.

Table S3. Descriptive Statistics

| Country | FDI | DI | EODB | GDP | TRA | NRT | XC | RQ | GE | CC |
| --- | --- | --- | --- | --- | --- | --- | --- | --- | --- | --- |
|  |  |  |  |  | Brazil |  |  |  |  |  |
| Mean | 3.160 | 18.482 | 55.524 | 1.139 | 26.214 | 33.336 | 2.704 | -0.001 | -0.233 | -0.208 |
| Maximum | 4.137 | 21.826 | 59.100 | 6.523 | 32.302 | 44.635 | 5.155 | 0.258 | -0.087 | 0.168 |
| Minimum | 1.734 | 14.626 | 54.527 | -4.358 | 22.106 | 18.499 | 1.673 | -0.293 | -0.520 | -0.559 |
| SD | 0.787 | 2.666 | 1.676 | 3.192 | 2.630 | 7.849 | 0.964 | 0.159 | 0.128 | 0.223 |
| Skewness | -0.683 | -0.027 | 1.504 | -0.315 | 0.596 | -0.356 | 1.018 | 0.013 | -0.950 | -0.115 |
| Kurtosis | 2.271 | 1.528 | 3.559 | 2.312 | 2.919 | 2.219 | 3.342 | 2.087 | 2.891 | 1.648 |
|  |  |  |  |  | China |  |  |  |  |  |
| Mean | 2.895 | 43.598 | 65.075 | 8.038 | 47.927 | 3.864 | 6.928 | -0.273 | 0.213 | -0.417 |
| Maximum | 4.554 | 46.660 | 78.163 | 13.636 | 64.479 | 9.648 | 8.277 | -0.179 | 0.644 | -0.071 |
| Minimum | 1.311 | 39.910 | 58.247 | 1.996 | 34.754 | 0.864 | 6.143 | -0.354 | -0.141 | -0.618 |
| SD | 1.141 | 2.254 | 8.792 | 2.762 | 10.211 | 2.641 | 0.683 | 0.058 | 0.237 | 0.152 |
| Skewness | 0.046 | -0.071 | 0.645 | 0.047 | 0.280 | 0.573 | 0.876 | 0.242 | 0.261 | 0.497 |
| Kurtosis | 1.630 | 1.834 | 1.459 | 3.127 | 1.704 | 2.375 | 2.570 | 1.726 | 1.883 | 2.462 |
|  |  |  |  |  | Egypt |  |  |  |  |  |
| Mean | 3.344 | 17.517 | 53.587 | 2.356 | 47.876 | 9.369 | 8.922 | -0.517 | -0.470 | -0.617 |
| Maximum | 9.349 | 22.390 | 60.100 | 5.078 | 71.681 | 15.582 | 17.783 | -0.148 | -0.208 | -0.465 |
| Minimum | -0.205 | 13.643 | 38.934 | -0.457 | 30.247 | 3.209 | 5.433 | -0.893 | -0.782 | -0.811 |
| SD | 2.673 | 2.485 | 7.674 | 1.726 | 12.471 | 4.033 | 4.778 | 0.248 | 0.169 | 0.097 |
| Skewness | 1.130 | 0.146 | -1.038 | -0.020 | 0.399 | -0.065 | 1.115 | 0.035 | -0.337 | -0.489 |
| Kurtosis | 3.330 | 2.190 | 2.462 | 2.183 | 1.978 | 1.861 | 2.447 | 1.658 | 2.144 | 2.415 |
|  |  |  |  |  | Ethiopia |  |  |  |  |  |
| Mean | 2.732 | 24.947 | 45.973 | 7.057 | 41.284 | 14.912 | 17.716 | -1.007 | -0.624 | -0.584 |
| Maximum | 5.576 | 40.671 | 48.208 | 10.357 | 55.601 | 27.811 | 34.927 | -0.848 | -0.442 | -0.390 |
| Minimum | 0.401 | 5.378 | 41.960 | 3.281 | 24.006 | 5.498 | 8.636 | -1.136 | -0.918 | -0.773 |
| SD | 1.681 | 13.718 | 1.857 | 1.824 | 9.419 | 6.267 | 7.988 | 0.081 | 0.146 | 0.122 |
| Skewness | 0.220 | -0.324 | -0.501 | -0.361 | -0.141 | 0.201 | 0.522 | 0.210 | -0.477 | 0.069 |
| Kurtosis | 1.887 | 1.276 | 2.274 | 2.785 | 1.934 | 2.494 | 2.393 | 2.298 | 2.326 | 1.678 |
|  |  |  |  |  | India |  |  |  |  |  |
| Mean | 1.858 | 35.390 | 55.371 | 4.677 | 45.769 | 3.385 | 55.460 | -0.337 | 0.013 | -0.390 |
| Maximum | 3.621 | 41.951 | 71.045 | 7.013 | 55.794 | 7.109 | 74.100 | -0.128 | 0.375 | -0.229 |
| Minimum | 0.766 | 28.752 | 50.559 | -6.726 | 37.504 | 1.748 | 41.349 | -0.481 | -0.222 | -0.541 |
| SD | 0.666 | 4.123 | 7.454 | 3.303 | 6.125 | 1.460 | 11.149 | 0.107 | 0.159 | 0.095 |
| Skewness | 0.806 | -0.117 | 1.347 | -2.613 | 0.365 | 0.872 | 0.248 | 0.411 | 0.551 | -0.006 |
| Kurtosis | 4.264 | 1.645 | 3.180 | 9.555 | 1.857 | 3.487 | 1.498 | 2.318 | 2.884 | 1.798 |
|  |  |  |  |  | Iran |  |  |  |  |  |
| Mean | 0.7548 | 37.4065 | 54.8000 | 0.7773 | 47.0553 | 25.2942 | 20631.88 | -1.4416 | -0.5338 | -0.7484 |
| Maximum | 1.5982 | 45.9988 | 58.6392 | 6.8357 | 58.5651 | 34.7791 | 42000.00 | -1.1746 | -0.2370 | -0.3592 |
| Minimum | 0.4573 | 29.1092 | 48.3608 | -4.9689 | 39.4226 | 13.1358 | 8613.989 | -1.7092 | -1.0171 | -1.1107 |
| SD | 0.3008 | 4.3438 | 3.6288 | 3.6481 | 5.3150 | 6.6576 | 13114.80 | 0.1591 | 0.1920 | 0.2125 |
| Skewness | 1.5951 | -0.0687 | -0.6608 | -0.0094 | 0.5224 | -0.1150 | 0.5693 | -0.0513 | -0.4473 | 0.0161 |
| Kurtosis | 4.9380 | 2.5285 | 1.9022 | 1.9597 | 2.4549 | 2.1397 | 1.6966 | 2.2074 | 3.7844 | 2.2195 |
|  |  |  |  |  | Russia |  |  |  |  |  |
| Mean | 2.356 | 22.631 | 67.789 | 2.603 | 50.042 | 13.684 | 41.948 | -0.371 | -0.366 | -0.965 |
| Maximum | 4.503 | 25.501 | 78.200 | 8.686 | 56.713 | 19.105 | 72.105 | -0.100 | 0.024 | -0.822 |
| Minimum | 0.503 | 18.926 | 62.277 | -7.828 | 45.967 | 7.588 | 24.853 | -0.580 | -0.602 | -1.141 |
| SD | 1.218 | 1.684 | 4.350 | 4.397 | 3.535 | 3.404 | 17.481 | 0.136 | 0.189 | 0.108 |
| Skewness | 0.035 | -0.481 | 1.454 | -0.586 | 0.701 | -0.292 | 0.612 | 0.367 | 0.616 | -0.189 |
| Kurtosis | 2.254 | 2.811 | 4.111 | 3.000 | 2.245 | 2.136 | 1.585 | 2.585 | 2.227 | 1.728 |
|  |  |  |  |  | South Africa | |  |  |  |  |
| Mean | 1.286 | 17.711 | 67.288 | 0.802 | 54.337 | 5.411 | 10.094 | 0.366 | 0.233 | 0.056 |
| Maximum | 3.127 | 21.287 | 71.440 | 4.591 | 65.975 | 11.990 | 16.459 | 0.747 | 0.567 | 0.484 |
| Minimum | 0.205 | 12.538 | 64.634 | -7.107 | 45.644 | 2.808 | 6.359 | -0.041 | 0.045 | -0.184 |
| SD | 0.823 | 1.914 | 2.078 | 2.886 | 4.847 | 2.149 | 3.370 | 0.262 | 0.156 | 0.206 |
| Skewness | 0.615 | -0.860 | 0.664 | -1.031 | 0.364 | 1.727 | 0.499 | -0.052 | 0.924 | 0.825 |
| Kurtosis | 2.538 | 4.541 | 2.462 | 4.447 | 3.427 | 6.188 | 1.773 | 1.723 | 3.051 | 2.490 |
|  |  |  |  |  | UAE |  |  |  |  |  |
| Mean | 3.447 | 23.241 | 73.238 | -1.864 | 151.227 | 21.617 | 3.673 | 0.759 | 1.142 | 1.060 |
| Max | 6.767 | 31.634 | 81.589 | 5.817 | 172.803 | 29.779 | 3.673 | 1.097 | 1.501 | 1.274 |
| Min | 0.447 | 18.771 | 65.032 | -17.145 | 116.623 | 11.806 | 3.673 | 0.319 | 0.688 | 0.886 |
| SD | 1.838 | 3.154 | 5.793 | 7.102 | 18.811 | 6.045 | 0.000 | 0.240 | 0.265 | 0.105 |
| Skewness | 0.461 | 0.858 | -0.085 | -0.932 | -0.722 | -0.373 | . | -0.212 | -0.205 | -0.023 |
| Kurtosis | 1.990 | 4.000 | 1.724 | 2.723 | 2.212 | 1.733 | . | 1.849 | 1.754 | 2.697 |
|  |  |  |  |  | Total |  |  |  |  |  |
| Mean | 2.426 | 26.769 | 59.849 | 2.843 | 56.859 | 14.541 | 2308.81 | -0.314 | -0.069 | -0.313 |
| Maximum | 9.349 | 46.660 | 81.589 | 13.636 | 172.803 | 44.635 | 42000.0 | 1.097 | 1.501 | 1.274 |
| Minimum | -0.205 | 5.378 | 38.934 | -17.145 | 22.106 | 0.864 | 1.673 | -1.709 | -1.017 | -1.141 |
| SD | 1.641 | 10.488 | 9.882 | 4.740 | 35.523 | 10.983 | 7768.42 | 0.650 | 0.554 | 0.582 |
| Skewness | 1.266 | 0.324 | 0.188 | -1.084 | 2.206 | 0.783 | 3.860 | -0.001 | 1.075 | 1.267 |
| Kurtosis | 5.481 | 1.896 | 2.361 | 5.534 | 6.860 | 2.716 | 17.602 | 2.650 | 3.737 | 3.977 |

NB: SD=Standard deviation
