# Supplementary material for: Investment attractiveness in BRICS+ economies: Evaluating business environment reforms, institutional quality, and macroeconomic factors
Source: PLoS One. 2025 Oct 16;20(10):e0334043. doi: 10.1371/journal.pone.0334043 (PMC12530542; doi:10.1371/journal.pone.0334043)
Supplement: S4 Table — (DOCX) [file pone.0334043.s004.docx]

## **S4 Table. Pearson Correlation Matrix Analysis**

The pairwise correlation analysis (See S4A Table) offers valuable insights into the strength and direction of linear relationships among various economic and institutional indicators under consideration. A detailed examination of these correlations can illuminate potential interdependencies, complementarities, and trade-offs, informing policy formulation and decision-making processes. This analysis unravels a complex web of associations among the variables under consideration, shedding light on the intricate interplay between various dimensions of economic variables, institutional quality, and the ease of doing business framework. Correlation coefficients range from -1 to +1, with higher absolute values indicating stronger associations. A coefficient of -1 indicates a perfect negative correlation, meaning that as one variable's value increases, the other variable's value decreases perfectly. Conversely, a coefficient of +1 indicates a perfect positive correlation, meaning that as one variable's value rises, the other variable's value also increases ideally. A coefficient of 0 suggests no linear association between the variables. The table below also includes p-values for each correlation coefficient. A low p-value (typically less than 0.05) indicates that the correlation is statistically significant and unlikely to be due to chance.

Financial Development (FID) has a high positive correlation with Economic Freedom (EFI) and a moderate positive correlation with Government Effectiveness (GE), suggesting that countries with better financial sector development tend to have higher levels of economic freedom and more effective governance. The Regulatory Quality (RQ) variable strongly correlates positively with Government Effectiveness (GE), indicating that countries with better regulatory practices tend to have more effective governance structures. Trade openness (TRA) has a moderate negative correlation with Exchange Rate (XC), suggesting that countries with higher trade openness tend to have more stable exchange rates. Domestic Investment (DI) has a moderate positive correlation with GDP growth (GDP), implying that higher domestic investment is associated with higher economic growth. The Rule of Law (RoL) variable has a moderate positive correlation with Government Effectiveness (GE), indicating that countries with stronger rule of law tend to have more effective governance. Inflation (INF) has a moderate negative correlation with Financial Development (FID), suggesting that countries with higher inflation tend to have less developed financial sectors. These correlations highlight the complex interplay between economic, institutional, and financial variables, underscoring the importance of strong governance, regulatory frameworks, and economic freedom in fostering financial sector development, domestic investment, and overall economic growth. The robustness check model in S4B Table corroborates and reinforces several key findings from the benchmark model. These insights underscore the multifaceted nature of economic development and the potential impact of various policy interventions and institutional reforms.

S4A Table. Pearson Correlation Matrix Analysis

| Variables | (1) | (2) | (3) | (4) | (5) | (6) | (7) | (8) | (9) | (10) | (11) | (12) | (13) |
| --- | --- | --- | --- | --- | --- | --- | --- | --- | --- | --- | --- | --- | --- |
| (1) FDI | 1.000 |  |  |  |  |  |  |  |  |  |  |  |  |
|  |  |  |  |  |  |  |  |  |  |  |  |  |  |
| (2) DI | -0.069 | 1.000 |  |  |  |  |  |  |  |  |  |  |  |
|  | (0.396) |  |  |  |  |  |  |  |  |  |  |  |  |
| (3) EDB | -0.159 | -0.069 | 1.000 |  |  |  |  |  |  |  |  |  |  |
|  | (0.050) | (0.400) |  |  |  |  |  |  |  |  |  |  |  |
| (4) GDP | 0.154 | 0.274* | -0.318* | 1.000 |  |  |  |  |  |  |  |  |  |
|  | (0.058) | (0.001) | (0.000) |  |  |  |  |  |  |  |  |  |  |
| (5) TRA | 0.188* | -0.085 | 0.501* | -0.270* | 1.000 |  |  |  |  |  |  |  |  |
|  | (0.020) | (0.299) | (0.000) | (0.001) |  |  |  |  |  |  |  |  |  |
| (6) NRT | 0.118 | -0.249* | -0.183* | -0.250* | 0.106 | 1.000 |  |  |  |  |  |  |  |
|  | (0.147) | (0.002) | (0.024) | (0.002) | (0.191) |  |  |  |  |  |  |  |  |
| (7) XC | -0.314* | 0.266* | -0.108 | -0.162* | -0.083 | 0.260* | 1.000 |  |  |  |  |  |  |
|  | (0.000) | (0.001) | (0.183) | (0.046) | (0.307) | (0.001) |  |  |  |  |  |  |  |
| (8) RQ | 0.251* | -0.344* | 0.616* | -0.201* | 0.572* | -0.068 | -0.503* | 1.000 |  |  |  |  |  |
|  | (0.002) | (0.000) | (0.000) | (0.013) | (0.000) | (0.400) | (0.000) |  |  |  |  |  |  |
| (9) GE | 0.078 | 0.014 | 0.704* | -0.222* | 0.773* | -0.116 | -0.245* | 0.805* | 1.000 |  |  |  |  |
|  | (0.337) | (0.863) | (0.000) | (0.006) | (0.000) | (0.152) | (0.002) | (0.000) |  |  |  |  |  |
| (10) CC | 0.142 | -0.157 | 0.470* | -0.291* | 0.762* | 0.119 | -0.263* | 0.776* | 0.868* | 1.000 |  |  |  |
|  | (0.079) | (0.053) | (0.000) | (0.000) | (0.000) | (0.144) | (0.001) | (0.000) | (0.000) |  |  |  |  |
| (11) EFI | 0.275* | -0.398* | 0.442* | -0.316* | 0.629* | 0.006 | -0.442* | 0.803* | 0.661* | 0.777* | 1.000 |  |  |
|  | (0.001) | (0.000) | (0.000) | (0.000) | (0.000) | (0.944) | (0.000) | (0.000) | (0.000) | (0.000) |  |  |  |
| (12) FID | 0.009 | 0.470* | 0.501* | 0.055 | 0.133 | -0.293* | -0.050 | 0.303* | 0.505* | 0.213* | 0.090 | 1.000 |  |
|  | (0.916) | (0.000) | (0.000) | (0.503) | (0.102) | (0.000) | (0.537) | (0.000) | (0.000) | (0.008) | (0.270) |  |  |
| (13) INF | -0.023 | 0.116 | -0.374* | -0.006 | -0.148 | 0.134 | 0.330* | -0.543* | -0.427* | -0.325* | -0.463* | -0.348* | 1.000 |
|  | (0.777) | (0.154) | (0.000) | (0.939) | (0.068) | (0.099) | (0.000) | (0.000) | (0.000) | (0.000) | (0.000) | (0.000) |  |

*Note: *** p<0.01, ** p<0.05, * p<0.1. EODB=Ease of Doing Business Aggregate. FDI = Foreign Direct Investment (% of GDP); DI =Domestic t Investment (% of GDP); TRA = Trade openness (% of GDP); XC=Exchange Rate (LCU per US$); RQ= regulatory Quality; CC= Control of Corruption; GE = Government Effectiveness; RoL = Rule of Law. EF=Economic Freedom (Index Value); FID=Financial Development (Domestic credit provided by financial sector (% of GDP), ES=Economic Stability Inflation, consumer prices (annual %) ( Source: World Bank and Heritage Foundation databases and Author's calculations*

S4B Table Pearson Correlation Matrix Analysis (Robustness)

| Variables | (1) | (2) | (3) | (4) | (5) | (6) | (7) | (8) | (9) | (10) | (11) | (12) | (13) | (14) | (15) | (16) | (17) | (18) | (19) | (20) | (21) | (22) | (23) |
| --- | --- | --- | --- | --- | --- | --- | --- | --- | --- | --- | --- | --- | --- | --- | --- | --- | --- | --- | --- | --- | --- | --- | --- |
| (1) FDI | 1.000 |  |  |  |  |  |  |  |  |  |  |  |  |  |  |  |  |  |  |  |  |  |  |
|  |  |  |  |  |  |  |  |  |  |  |  |  |  |  |  |  |  |  |  |  |  |  |  |
| (2) DI | -0.069 | 1.000 |  |  |  |  |  |  |  |  |  |  |  |  |  |  |  |  |  |  |  |  |  |
|  | (0.396) |  |  |  |  |  |  |  |  |  |  |  |  |  |  |  |  |  |  |  |  |  |  |
| (3) EDB | -0.159 | -0.069 | 1.000 |  |  |  |  |  |  |  |  |  |  |  |  |  |  |  |  |  |  |  |  |
|  | (0.050) | (0.400) |  |  |  |  |  |  |  |  |  |  |  |  |  |  |  |  |  |  |  |  |  |
| (4) SB | -0.204* | -0.099 | 0.832* | 1.000 |  |  |  |  |  |  |  |  |  |  |  |  |  |  |  |  |  |  |  |
|  | (0.012) | (0.221) | (0.000) |  |  |  |  |  |  |  |  |  |  |  |  |  |  |  |  |  |  |  |  |
| (5) DCP | -0.139 | -0.484* | 0.585* | 0.464* | 1.000 |  |  |  |  |  |  |  |  |  |  |  |  |  |  |  |  |  |  |
|  | (0.088) | (0.000) | (0.000) | (0.000) |  |  |  |  |  |  |  |  |  |  |  |  |  |  |  |  |  |  |  |
| (6) GEL | 0.233* | -0.132 | 0.392* | 0.263* | 0.383* | 1.000 |  |  |  |  |  |  |  |  |  |  |  |  |  |  |  |  |  |
|  | (0.004) | (0.105) | (0.000) | (0.001) | (0.000) |  |  |  |  |  |  |  |  |  |  |  |  |  |  |  |  |  |  |
| (7) RP | -0.015 | 0.102 | 0.714* | 0.603* | 0.440* | 0.434* | 1.000 |  |  |  |  |  |  |  |  |  |  |  |  |  |  |  |  |
|  | (0.852) | (0.208) | (0.000) | (0.000) | (0.000) | (0.000) |  |  |  |  |  |  |  |  |  |  |  |  |  |  |  |  |  |
| (8) GC | -0.468* | -0.015 | 0.565* | 0.592* | 0.156 | -0.011 | 0.161* | 1.000 |  |  |  |  |  |  |  |  |  |  |  |  |  |  |  |
|  | (0.000) | (0.852) | (0.000) | (0.000) | (0.055) | (0.894) | (0.047) |  |  |  |  |  |  |  |  |  |  |  |  |  |  |  |  |
| (9) PIM | -0.171* | -0.147 | 0.621* | 0.541* | 0.169* | 0.071 | 0.076 | 0.763* | 1.000 |  |  |  |  |  |  |  |  |  |  |  |  |  |  |
|  | (0.034) | (0.069) | (0.000) | (0.000) | (0.036) | (0.380) | (0.350) | (0.000) |  |  |  |  |  |  |  |  |  |  |  |  |  |  |  |
| (10) PT | -0.148 | -0.148 | 0.606* | 0.409* | 0.602* | 0.185* | 0.697* | 0.197* | 0.114 | 1.000 |  |  |  |  |  |  |  |  |  |  |  |  |  |
|  | (0.068) | (0.068) | (0.000) | (0.000) | (0.000) | (0.022) | (0.000) | (0.015) | (0.161) |  |  |  |  |  |  |  |  |  |  |  |  |  |  |
| (11) TAB | 0.147 | 0.183* | 0.618* | 0.513* | 0.200* | 0.480* | 0.479* | 0.319* | 0.444* | 0.188* | 1.000 |  |  |  |  |  |  |  |  |  |  |  |  |
|  | (0.069) | (0.023) | (0.000) | (0.000) | (0.013) | (0.000) | (0.000) | (0.000) | (0.000) | (0.020) |  |  |  |  |  |  |  |  |  |  |  |  |  |
| (12) EC | -0.038 | 0.087 | 0.458* | 0.351* | 0.286* | -0.031 | 0.576* | -0.044 | -0.051 | 0.461* | -0.044 | 1.000 |  |  |  |  |  |  |  |  |  |  |  |
|  | (0.643) | (0.284) | (0.000) | (0.000) | (0.000) | (0.703) | (0.000) | (0.588) | (0.530) | (0.000) | (0.593) |  |  |  |  |  |  |  |  |  |  |  |  |
| (13) RI | 0.036 | -0.077 | 0.590* | 0.584* | 0.125 | 0.006 | 0.451* | 0.334* | 0.474* | 0.158 | 0.345* | 0.544* | 1.000 |  |  |  |  |  |  |  |  |  |  |
|  | (0.658) | (0.343) | (0.000) | (0.000) | (0.124) | (0.939) | (0.000) | (0.000) | (0.000) | (0.051) | (0.000) | (0.000) |  |  |  |  |  |  |  |  |  |  |  |
| (14) GDP | 0.154 | 0.274* | -0.318* | -0.304* | -0.470* | -0.230* | -0.194* | -0.230* | -0.206* | -0.215* | -0.300* | 0.056 | -0.066 | 1.000 |  |  |  |  |  |  |  |  |  |
|  | (0.058) | (0.001) | (0.000) | (0.000) | (0.000) | (0.004) | (0.016) | (0.004) | (0.011) | (0.008) | (0.000) | (0.490) | (0.418) |  |  |  |  |  |  |  |  |  |  |
| (15) TRA | 0.188* | -0.085 | 0.501* | 0.262* | 0.470* | 0.563* | 0.627* | -0.001 | 0.119 | 0.673* | 0.455* | 0.122 | -0.072 | -0.270* | 1.000 |  |  |  |  |  |  |  |  |
|  | (0.020) | (0.299) | (0.000) | (0.001) | (0.000) | (0.000) | (0.000) | (0.990) | (0.143) | (0.000) | (0.000) | (0.132) | (0.374) | (0.001) |  |  |  |  |  |  |  |  |  |
| (16) NRT | 0.118 | -0.249* | -0.183* | -0.205* | 0.093 | 0.152 | 0.081 | -0.304* | -0.322* | -0.135 | -0.097 | 0.048 | -0.051 | -0.250* | 0.106 | 1.000 |  |  |  |  |  |  |  |
|  | (0.147) | (0.002) | (0.024) | (0.011) | (0.251) | (0.060) | (0.317) | (0.000) | (0.000) | (0.097) | (0.235) | (0.552) | (0.529) | (0.002) | (0.191) |  |  |  |  |  |  |  |  |
| (17) XC | -0.314* | 0.266* | -0.108 | -0.069 | 0.123 | -0.140 | 0.000 | -0.015 | -0.194* | -0.100 | -0.165* | 0.052 | -0.236* | -0.162* | -0.083 | 0.260* | 1.000 |  |  |  |  |  |  |
|  | (0.000) | (0.001) | (0.183) | (0.398) | (0.130) | (0.083) | (0.995) | (0.857) | (0.016) | (0.220) | (0.042) | (0.527) | (0.003) | (0.046) | (0.307) | (0.001) |  |  |  |  |  |  |  |
| (18) RQ | 0.251* | -0.344* | 0.616* | 0.400* | 0.327* | 0.374* | 0.354* | 0.294* | 0.630* | 0.377* | 0.631* | 0.033 | 0.375* | -0.201* | 0.572* | -0.068 | -0.503* | 1.000 |  |  |  |  |  |
|  | (0.002) | (0.000) | (0.000) | (0.000) | (0.000) | (0.000) | (0.000) | (0.000) | (0.000) | (0.000) | (0.000) | (0.681) | (0.000) | (0.013) | (0.000) | (0.400) | (0.000) |  |  |  |  |  |  |
| (19) GE | 0.078 | 0.014 | 0.704* | 0.392* | 0.393* | 0.487* | 0.515* | 0.271* | 0.530* | 0.521* | 0.677* | 0.108 | 0.202* | -0.222* | 0.773* | -0.116 | -0.245* | 0.805* | 1.000 |  |  |  |  |
|  | (0.337) | (0.863) | (0.000) | (0.000) | (0.000) | (0.000) | (0.000) | (0.001) | (0.000) | (0.000) | (0.000) | (0.183) | (0.012) | (0.006) | (0.000) | (0.152) | (0.002) | (0.000) |  |  |  |  |  |
| (20) CC | 0.142 | -0.157 | 0.470* | 0.188* | 0.411* | 0.399* | 0.334* | 0.062 | 0.338* | 0.458* | 0.555* | -0.055 | -0.031 | -0.291* | 0.762* | 0.119 | -0.263* | 0.776* | 0.868* | 1.000 |  |  |  |
|  | (0.079) | (0.053) | (0.000) | (0.020) | (0.000) | (0.000) | (0.000) | (0.447) | (0.000) | (0.000) | (0.000) | (0.502) | (0.707) | (0.000) | (0.000) | (0.144) | (0.001) | (0.000) | (0.000) |  |  |  |  |
| (21) EFI | 0.275* | -0.398* | 0.442* | 0.257* | 0.367* | 0.292* | 0.337* | 0.086 | 0.350* | 0.456* | 0.533* | -0.099 | 0.132 | -0.316* | 0.629* | 0.006 | -0.442* | 0.803* | 0.661* | 0.777* | 1.000 |  |  |
|  | (0.001) | (0.000) | (0.000) | (0.001) | (0.000) | (0.000) | (0.000) | (0.290) | (0.000) | (0.000) | (0.000) | (0.222) | (0.105) | (0.000) | (0.000) | (0.944) | (0.000) | (0.000) | (0.000) | (0.000) |  |  |  |
| (22) FID | 0.009 | 0.470* | 0.501* | 0.299* | -0.105 | 0.076 | 0.348* | 0.202* | 0.392* | 0.035 | 0.525* | 0.383* | 0.520* | 0.055 | 0.133 | -0.293* | -0.050 | 0.303* | 0.505* | 0.213* | 0.090 | 1.000 |  |
|  | (0.916) | (0.000) | (0.000) | (0.000) | (0.197) | (0.347) | (0.000) | (0.012) | (0.000) | (0.671) | (0.000) | (0.000) | (0.000) | (0.503) | (0.102) | (0.000) | (0.537) | (0.000) | (0.000) | (0.008) | (0.270) |  |  |
| (23) INF | -0.023 | 0.116 | -0.374* | -0.270* | -0.043 | -0.136 | -0.202* | -0.201* | -0.402* | -0.112 | -0.456* | 0.034 | -0.397* | -0.006 | -0.148 | 0.134 | 0.330* | -0.543* | -0.427* | -0.325* | -0.463* | -0.348* | 1.000 |
|  | (0.777) | (0.154) | (0.000) | (0.001) | (0.595) | (0.094) | (0.012) | (0.013) | (0.000) | (0.167) | (0.000) | (0.680) | (0.000) | (0.939) | (0.068) | (0.099) | (0.000) | (0.000) | (0.000) | (0.000) | (0.000) | (0.000) |  |
|  | | | | | | | | | | | | | | | | | | | | | | | |

*Note: *** p<0.01, ** p<0.05, * p<0.1. EODB=Ease of Doing Business Aggregate. SB = Starting a Business; DCP = Dealing with Construction Permits; GEL = Getting Electricity; RP = Registering Property; GC = Getting Credit; PI = Protecting Minority Investors; TAB = Trading Across Borders; EC = Enforcing Contracts; RI = Resolving Insolvency; PT = Paying Taxes;* *FDI = Foreign Direct Investment (% of GDP); DI =Domestic t Investment (% of GDP); TRA = Trade openness (% of GDP); XC=Exchange Rate( LCU per US$); RQ= regulatory Quality; CC= Control of Corruption; GE = Government Effectiveness; RoL = Rule of Law. EF=Economic Freedom (Index Value); FID=Financial Development (Domestic credit provided by financial sector (% of GDP), ES=Economic Stability* *Inflation, consumer prices (annual %) ( Source: World Bank and Heritage Foundation databases and Author's calculations.*
