# Supplementary material for: Investment attractiveness in BRICS+ economies: Evaluating business environment reforms, institutional quality, and macroeconomic factors
Source: PLoS One. 2025 Oct 16;20(10):e0334043. doi: 10.1371/journal.pone.0334043 (PMC12530542; doi:10.1371/journal.pone.0334043)
Supplement: S5 Table — (DOCX) [file pone.0334043.s005.docx]

## S5 Table. Cross-Sectional Dependence Test

The Cross-Sectional Dependence (CSD) test is a crucial diagnostic tool in panel data analysis, designed to evaluate the existence of cross-sectional dependence or contemporaneous correlation among the cross-sectional units. Ignoring such dependencies can lead to skewed inferences and unreliable estimation outcomes. S5 Table offers an in-depth analysis of cross-sectional dependence across the variables under study. The results indicate each variable's CD test statistic and corresponding p-value. The null hypothesis for this test is cross-sectional independence, with p-values near zero indicating the presence of cross-sectional dependence. The results reveal that variables such as EDB, SB, DCP, RP, GC, PIM, GDP, NRT, and XC display statistically significant cross-sectional dependence, as their p-values are close to zero. This suggests that the observations for these variables are not independent across the cross-sectional units, implying the existence of common shocks, spillover effects, or unobserved factors influencing multiple cross-sectional units simultaneously. The average joint T, mean ρ, and mean abs(ρ) provide further insights into the extent of cross-sectional dependence. The average joint T represents the average number of cross-sectional units for which the null hypothesis of no cross-sectional dependence is rejected. The mean ρ and mean abs(ρ) indicate the average and absolute average values of the pairwise correlation coefficients, respectively. Variables with higher mean abs(ρ) values, such as PIM (0.68), NRT (0.6), XC (0.59), and SB (0.57), exhibit stronger cross-sectional dependence**.**

To further validate the presence of cross-sectional dependence, robust tests, including the Breusch-Pagan LM, Pesaran scaled LM, and Pesaran CD tests, are presented in the lower part of Table S6. Both the Breusch-Pagan LM and Pesaran scaled LM tests reject the null hypothesis of cross-sectional independence, reinforcing the evidence of cross-sectional dependence in the data. However, the Pesaran CD test fails to reject the null hypothesis of cross-sectional independence at conventional significance levels. It's important to note that this test may have lower power in detecting cross-sectional dependence when the number of cross-sectional units is small or when the dependence pattern is complex or heterogeneous across units. The results of the cross-sectional dependence analysis warrant using second-generation tests to account for the cross-sectional dependencies in panel data models.

S5 Table. Cross-Sectional Dependence Test

| Variable | CD-test | p-value | average joint T | mean ρ | mean abs(ρ) |
| --- | --- | --- | --- | --- | --- |
| FDI | -0.041 | 0.968 | 17 | 0 | 0.34 |
| DI | 2.024 | 0.043 | 17 | 0.08 | 0.3 |
| EDB | 12.718 | 0.000 | 17 | 0.51 | 0.56 |
| SB | 14.045 | 0.000 | 17 | 0.57 | 0.57 |
| DCP | 12.817 | 0.000 | 17 | 0.52 | 0.57 |
| GEL | 0.736 | 0.462 | 17 | 0.03 | 0.55 |
| RP | 2.745 | 0.006 | 17 | 0.11 | 0.4 |
| GC | 3.81 | 0.000 | 17 | 0.15 | 0.52 |
| PIM | 8.128 | 0.000 | 17 | 0.33 | 0.68 |
| PT | 0.021 | 0.983 | 17 | 0 | 0.37 |
| TAB | 3.447 | 0.001 | 17 | 0.14 | 0.41 |
| EC | -2.469 | 0.014 | 17 | -0.1 | 0.83 |
| RI | -1.262 | 0.207 | 17 | -0.05 | 0.48 |
| GDP | 8.018 | 0.000 | 17 | 0.32 | 0.4 |
| TRA | 1.445 | 0.148 | 17 | 0.06 | 0.41 |
| NRT | 14.569 | 0.000 | 17 | 0.59 | 0.6 |
| XC | 11.15 | 0.000 | 17 | 0.45 | 0.59 |
| RQ | -0.151 | 0.88 | 17 | -0.01 | 0.41 |
| GE | -0.909 | 0.363 | 17 | -0.04 | 0.38 |
| CC | -0.999 | 0.318 | 17 | -0.04 | 0.38 |
| Test | | Statistic | | Prob. | |
| Breusch-Pagan LM | | 93.058 | | 0.000 | |
| Pesaran scaled LM | | 6.724 | | 0.000 | |
| Pesaran CD | | -1.027 | | 0.304 | |

*Under the null hypothesis of cross-section independence, CD Can you please provide me with access to this document? N(0,1) P-values close to zero indicate data are correlated across panel groups. GDP=GDP Growth (annual %); SB = Starting a Business; DCP = Dealing with Construction Permits; GE = Getting Electricity; RP = Registering Property; GC = Getting Credit; PIM = Protecting Minority Investors; TAB = Trading Across Borders; EC = Enforcing Contracts; RI = Resolving Insolvency; PT = Paying Taxes; FDI = Foreign Direct Investment (% of GDP); DI= Domestic Investment, TRA= Trade openness (% of GDP); XC=Exchange rate, NRT= Natural Resource, RQ=Regulatory Quality GE=Governance Effectiveness,*

*CC=Control of Corruption. Source: World Bank database and Author's calculations. *, **, *** represent 1%,5% and 10% significance level*
