# Supplementary material for: Investment attractiveness in BRICS+ economies: Evaluating business environment reforms, institutional quality, and macroeconomic factors
Source: PLoS One. 2025 Oct 16;20(10):e0334043. doi: 10.1371/journal.pone.0334043 (PMC12530542; doi:10.1371/journal.pone.0334043)
Supplement: S6 Table — (DOCX) [file pone.0334043.s006.docx]

## S6 Table. Variance Inflation Factor Analysis and Slope Heterogeneity Tests

S6 Table provides the results of a Variance Inflation Factor (VIF) test, a statistical tool used to detect multicollinearity. The test is conducted for the basic model. Multicollinearity explains the situation where predictor variables in a regression model are highly interrelated. This condition can complicate the interpretation of a regression analysis, as it may lead to less precise estimates of the coefficients and their standard errors, thereby obscuring the individual impacts of each predictor variable. In these results, the VIF values for the variables EDB, TRA, GDP, NRT, and XC range from 1.11 to 1.59. As a general guideline, a VIF value below 5 or 10 is usually considered acceptable, indicating that the predictor variables are not overly correlated. The mean VIF value in this case is 1.34, which is relatively low, suggesting that multicollinearity is not a significant issue in this particular model. Therefore, the estimates produced by this model should be reliable, and the individual effects of each predictor variable can be interpreted with a reasonable degree of confidence. This is a positive indication, suggesting that the model is robust to the potential pitfalls of multicollinearity. Also, the lower part of the table outlines the results of a heterogeneity test conducted on a panel data model. The null hypothesis of this test asserts that the slope coefficients are homogeneous across the panel. However, both the Non-adjusted Delta ($\widehat{\Delta}$) and Adjusted Delta ($\widehat{\text{Δ}}_{\text{adj}}$) test statistics reject the null hypothesis at a 1% significance level. Specifically, the p-value for the Non-adjusted Delta ($\widehat{\Delta}$) test is less than 0.001, and for the Adjusted Delta ($\widehat{\text{Δ}}_{\text{adj}}$) test, it is less than 0.000. These findings lead to the conclusion of slope heterogeneity existence, suggesting that the dependent and independent variables vary across different groups within the panel. Moreover, these results confirm the presence of cross-dependency among the countries under study, implying that the economic dynamics in one country could potentially impact those in another, highlighting the interconnected nature of these economies.

S6 Table. Variance Inflation Factor Analysis and Slope Heterogeneity Tests

| Variable | VIF | 1/VIF |
| --- | --- | --- |
| EDB | 1.59 | 0.628 |
| TRA | 1.43 | 0.698 |
| GDP | 1.28 | 0.780 |
| NRT | 1.27 | 0.790 |
| XC | 1.11 | 0.900 |
| Mean VIF | 1.34 |  |
| Slope Heterogeneity | Test Statistics | P value |
| Non-adjusted Delta test $\widehat{\Delta}$ | 3.356 | 0.001 |
| Adjusted Delta test $\widehat{\text{Δ}}_{\text{adj}}$ | 4.376 | 0.000 |

*NB: For the Slope Heterogeneity, Variables partialled out: constant. H0: slope coefficients are homogenous*
