# Supplementary material for: Investment attractiveness in BRICS+ economies: Evaluating business environment reforms, institutional quality, and macroeconomic factors
Source: PLoS One. 2025 Oct 16;20(10):e0334043. doi: 10.1371/journal.pone.0334043 (PMC12530542; doi:10.1371/journal.pone.0334043)
Supplement: S7 Table — (DOCX) [file pone.0334043.s007.docx]

## S7 Table. Panel Unit Root Tests

Unit root tests are essential in time series analysis to determine whether variables exhibit stationarity. Stationarity means that statistical properties (such as mean and variance) remain constant over time. Non-stationary variables can lead to misleading regression results. In the S7 Table, we applied the CIPS and Maddala-Wu tests to variables at both the level and first difference, with and without a deterministic trend. Most variables show non-stationarity at the levels. However, exceptions, like EDB, SB, DCP, and CC, suggest stationarity at specific lag lengths. When testing first differences, the null hypothesis of a unit root is overwhelmingly rejected, indicating that these variables are integrated of order one (I(1)). We use first differences for modeling to avoid spurious correlations, emphasizing the importance of pre-processing steps and proper consideration of data properties.

S7 Table. Panel Unit Root Tests

|  | | Levels Estimations | | | | I^st^ Difference Estimations | | | |
| --- | --- | --- | --- | --- | --- | --- | --- | --- | --- |
|  | | Maddala and Wu | | CIPS | | Maddala and Wu | | CIPS | |
| Variables | lags | Without Trend | With Trend | Without Trend | With Trend | Without Trend | With Trend | Without Trend | With Trend |
| FDI | 0 | 40.752* | 42.999* | -2.816* | -2.089* | 147.11*4 | 107.48* | -6* | -4.349* |
|  | 1 | 42.56* | 44.514* | -2.678* | -1.219 | 96.053* | 77.782* | -3.525* | -1.974** |
|  | 2 | 42.168* | 60.504* | -0.35 | 0.525 | 54.107* | 36.41* | -1.232 | -0.433 |
| DI | 0 | 20.344 | 13.784 | 0.128 | 0.717 | 80.985* | 61.836* | -4.077* | -2.618* |
|  | 1 | 33.348* | 23.598 | 0.224 | 0.665 | 42.832* | 29.691** | -0.943* | 0.58* |
|  | 2 | 48.588* | 39.025* | -0.855 | 0.958 | 42.129* | 26.43* | -0.091 | 1.517** |
| EDB | 0 | 9.085 | 7.343 | 2.479 | 3.053 | 63.497* | 43.838* | -3.525 | -3.43* |
|  | 1 | 18.198 | 13.564 | 2.266 | 3.776 | 39.046* | 36.173** | -0.473 | -1.928** |
|  | 2 | 49.094* | 30.429** | 3.805 | 4.96 | 39.084* | 28.682 | 1.917 | -2.403* |
| SB | 0 | 8.226 | 3.16 | 0.033 | 2.807 | 104.917* | 108.086* | -5.077* | -5.202* |
|  | 1 | 11.422 | 3.441 | 1.017 | 3.356 | 24.464 | 23.538 | 0.947 | 0.504 |
|  | 2 | 15.653 | 6.551 | 1.911 | 4.692 | 16.567 | 18.18 | 1.942 | -0.067 |
| DCP | 0 | 11.807 | 11.416 | 1.837 | 2.762 | 117.648* | 111.013* | -4.744* | -4.963* |
|  | 1 | 19.569 | 7.629 | 2.67 | 2.877 | 39.945* | 39.481* | 0.156 | -0.261 |
|  | 2 | 34.448* | 18.89 | 2.868 | 2.848 | 22.925 | 20.468 | 0.381 | -0.022 |
| GEL | 0 | 20.808 | 25.384 | 3.418 | 1.907 | 126.96* | 95.147* | -5.059* | -5.56* |
|  | 1 | 11.518 | 19.342 | 4.049 | 3.369 | 82.657* | 55.562* | -0.631 | -4.403* |
|  | 2 | 7.486 | 6.547 | 1.732 | 3.706 | 21.875 | 18.223 | 2.898 | 1.498 |
| RP | 0 | 16.839 | 7.736 | 0.628 | -0.118 | 101.17* | 80.224* | -5.74* | -4.729* |
|  | 1 | 18.181 | 8.616 | 1.057 | 0.666 | 42.911* | 30.56** | -3.382* | -3.087* |
|  | 2 | 22.602 | 10.633 | 0.435 | 2.848 | 14.631 | 6.435 | 1.276 | 2.567 |
| GC | 0 | 6.604 | 5.92 | 2.93 | 0.956 | 98.748* | 76.575* | -5.981* | -5.029* |
|  | 1 | 7.128 | 6.405 | 4.554 | 1.008 | 47.607* | 34.72* | -0.654 | 0.278 |
|  | 2 | 7.786 | 7.461 | 4.858 | 1.393 | 30.862** | 24.504 | -1.57** | 1.054 |
| PIM | 0 | 1.542 | 24.809 | -1.682** | -0.6 | 96.319* | 64.563* | -5.895* | -4.679* |
|  | 1 | 1.43 | 5.569 | 2.535 | 3.573 | 27.202*** | 13.175 | 0.98 | 1.564 |
|  | 2 | 2.214 | 10.087 | 2.891 | 3.805 | 16.675 | 5.242 | 2.293 | 3.202 |
| PT | 0 | 6.583 | 3.959 | 3.988 | 3.678 | 73.355* | 55.934* | -2.489* | -2.255 |
|  | 1 | 9.772 | 5.784 | 3.147 | 1.376 | 33.771** | 26.783*** | -0.429 | 0.562 |
|  | 2 | 12.816 | 7.01 | 1.906 | 2.182 | 26.384*** | 30.348 | 1.066 | 1.677 |
| TAB | 0 | 7.385 | 6.815 | 2.051 | 2.4 | 95.905* | 71.874* | -4.418* | -5.117* |
|  | 1 | 15.489 | 9.011 | 2.21 | 3.25 | 26.583** | 12.156 | 0.363 | 0.245 |
|  | 2 | 23.883 | 15.138 | 1.951 | 3.872 | 18.177 | 6.903 | 2.585 | 2.179 |
| EC | 0 | 4.026 | 7.039 | 0.27 | 1.034 | 101.211* | 69.562* | -3.205* | -1.678** |
|  | 1 | 4.287 | 7.32 | 0.472 | 0.602 | 35.844* | 16.961 | -2.23* | -0.394 |
|  | 2 | 5.021 | 7.812 | 2.222 | 3.087 | 20.008 | 7.372 | 1.663 | 3.907 |
| RI | 0 | 5.261 | 8.118 | 1.339 | 3.727 | 72.479* | 67.077* | -3.424* | -2.897* |
|  | 1 | 11.928 | 13.499 | 3.063 | 5.145 | 38.294* | 29.28** | 1.095 | 1.489 |
|  | 2 | 5.501 | 6.453 | 5.885 | 7.288 | 37.764* | 75.318* | 3.418 | 1.933 |
| GDP | 0 | 27.539 | 40* | -1.109 | 0.021 | 135.94* | 94.258* | -5.433* | -3.581* |
|  | 1 | 17.448 | 35.022* | -0.345 | 2.088 | 91.221* | 60.031* | -0.885 | 1.067 |
|  | 2 | 15.262 | 20.049 | 0.326 | 2.952 | 67.393* | 41.564* | 0.294 | 2.216 |
| TRA | 0 | 18.612 | 15.467 | 1.987 | 1.712 | 116.769* | 96.931* | -4.471* | -3.706* |
|  | 1 | 25.961 | 21.405 | 1.559 | 2.434 | 68.782* | 65.971* | -0.976 | 1.328*** |
|  | 2 | 27.433*** | 12.617 | 0.954 | 2.351 | 50.636* | 56.824* | 0.116 | -0.57* |
| NRT | 0 | 15.808 | 41.584* | -0.443 | -0.248 | 159.697* | 116.689* | -4.393* | -2.47* |
|  | 1 | 12.569 | 37.665* | -2.329* | -1.989** | 129.222* | 92.989* | -4.059* | -2.281* |
|  | 2 | 8.273 | 16.77 | 1.297 | 2.381 | 35.495* | 16.039 | -0.349 | 1.8 |
| XC | 0 | 4.232 | 5.245 | 0.945 | 0.978 | 37.613* | 34.517* | -0.905 | -0.585 |
|  | 1 | 5.903 | 8.557 | -0.925 | -0.658 | 31.062** | 30.803** | -3.191* | -3.278* |
|  | 2 | 5.849 | 7.414 | 0.461 | 0.823 | 17.69 | 18.235 | 0.32 | -0.615 |
| RQ | 0 | 18.322 | 34.822* | -0.537 | 0.321 | 153.285* | 122.475* | -5.201* | -3.638* |
|  | 1 | 22.337 | 26.434*** | -1.35*** | 0.837 | 64.363* | 39.569* | -0.642 | 1.176 |
|  | 2 | 18.721 | 14.568 | -1.138 | 1.542 | 21.413 | 15.661 | 0.941 | 2.573 |
| GE | 0 | 16.357 | 13.022 | 1.313 | 1.578 | 130.107* | 100.581* | -5.341* | -4.083* |
|  | 1 | 21.632 | 27.143*** | 1.95 | 1.981 | 62.946* | 42.614* | -2.216* | -0.899 |
|  | 2 | 13.667 | 16.641 | 2.438 | 2.995 | 40.331* | 27.218*** | -0.323 | -0.515 |
| CC | 0 | 13.016 | 12.058 | 1.031 | -1.59*** | 107.127* | 80.561* | -6.45* | -5.08* |
|  | 1 | 18.676 | 16.441 | 1.186 | 1.587*** | 43.797* | 31.814** | -3.695* | -2.975* |
|  | 2 | 23.101 | 31.852** | 0.389 | -1.36*** | 27.187*** | 12.205 | -1.407* | 0.939 |

*NB: *, ** and ***denote 1, 5 and 10 percentage significance levels, respectively*
