# Supplementary material for: Investment attractiveness in BRICS+ economies: Evaluating business environment reforms, institutional quality, and macroeconomic factors
Source: PLoS One. 2025 Oct 16;20(10):e0334043. doi: 10.1371/journal.pone.0334043 (PMC12530542; doi:10.1371/journal.pone.0334043)
Supplement: S8 Table — (DOCX) [file pone.0334043.s008.docx]

## S8 Table. Panel Cointegration Test

S8 Table outlines the results from the Westerlund ECM panel cointegration tests, which are utilized to check for a long-term equilibrium relationship among variables in panel data. These tests generate four statistics: Gt, Ga, Pt, and Pa, each with its value, Z-value, P-value, and robust P-value. The robust P-value, which is adjusted for serial correlation and cross-sectional dependence, is considered a more accurate indicator of statistical significance than the standard P-value. The hypothesis tested is that no cointegration exists among the variables, meaning they do not have a long-term equilibrium relationship. While the Gt statistic supports this hypothesis for the FDI model, the Ga, Pt, and Pa statistics contradict it, indicating cointegration as per their robust P-values, suggesting that despite some conflicting evidence, there is likely a long-term equilibrium relationship among the variables.

In contrast, all four statistics are negative for the domestic investment model, and their robust p-values exceed 0.05, pointing to a lack of solid evidence for cointegration within this model. The PMG estimator does not require a pre-test for cointegration. The PMG estimator inherently allows for different short-run dynamics and error correction terms across groups while constraining the long-run coefficients to be the same. This flexibility means that the PMG model can accommodate situations where the variables are not necessarily cointegrated in the conventional sense.

S8 Table. Westerlund ECM Panel Cointegration Tests

| Statistic | Value | Z-value | P-value | Robust P-value |
| --- | --- | --- | --- | --- |
| FDI Model | | | | |
| Gt | -2.336 | 0.406 | 0.342 | 0.203 |
| Ga | -4.605 | 2.781 | 0.997 | 0.013 |
| Pt | -6.832 | 0.972 | 0.166 | 0.083 |
| Pa | -5.062 | 1.12 | 0.869 | 0.005 |
| Domestic Investment Model | | | | |
| Gt | -1.967 | 0.035 | 0.514 | 0.165 |
| Ga | -3.073 | 2.871 | 0.998 | 0.623 |
| Pt | -7.767 | -2.302 | 0.011 | 0.068 |
| Pa | -4.034 | 0.854 | 0.803 | 0.31 |

Note: H0: no cointegration
