# Supplementary material for: Investment attractiveness in BRICS+ economies: Evaluating business environment reforms, institutional quality, and macroeconomic factors
Source: PLoS One. 2025 Oct 16;20(10):e0334043. doi: 10.1371/journal.pone.0334043 (PMC12530542; doi:10.1371/journal.pone.0334043)
Supplement: S1 File — (DOCX) [file pone.0334043.s009.docx]

## S1 Figure–S11 Figure. Average Values for Ease of Doing Business Index and its Indicators

S1 Fig–S11 Fig. offer an in-depth look at the central tendencies of the Ease of Doing Business (EDB) indicators, shedding light on their average trends and offering insights into the regulatory landscape. The overall EDB score, reflecting the regulatory environment’s supportiveness for businesses, has seen a steady rise from 54.49 in 2004 to 66.71 in 2020, indicating ongoing regulatory improvements and policy efforts to create a more favorable business setting. The mean score for ‘Dealing with Construction Permits’ has notably increased from 42.76 to 71.91 over the same period, signifying streamlined regulations and reduced administrative hurdles in construction. The ‘Getting Electricity’ indicator has maintained high average scores, climbing from 70.27 to 80.49, highlighting continuous advancements in ensuring reliable and affordable electricity, which is vital for a supportive business environment. Other indicators like ‘Registering Property,’ ‘Getting Credit,’ ‘Protecting Minority Investors,’ ‘Trading Across Borders,’ and ‘Enforcing Contracts’ have shown improvements at varying rates, suggesting some areas may still benefit from further reforms. The ‘Paying Taxes’ indicator has seen a slight rise in its average score from 65.33 to 67.05, pointing to ongoing challenges in tax compliance and the need for more streamlined tax procedures. The ‘Resolving Insolvency’ indicator has recorded modest increases in its mean score from 42.52 to 47.48, indicating ongoing complexities in insolvency proceedings and the need for more effective reform measures. The analysis of EDB scores and their components indicates significant strides in regulatory reform across various sectors. However, areas like insolvency resolution and tax payments still require focused efforts to improve the business climate further. It’s important to recognize that these averages may conceal variations across different countries or regions, suggesting a need for detailed country-specific analyses to address particular challenges and craft appropriate policy solutions.


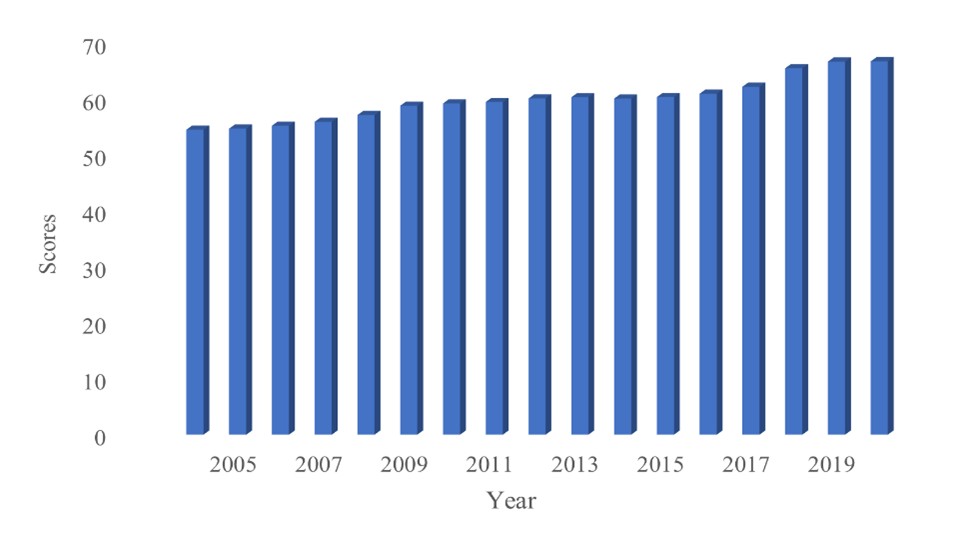

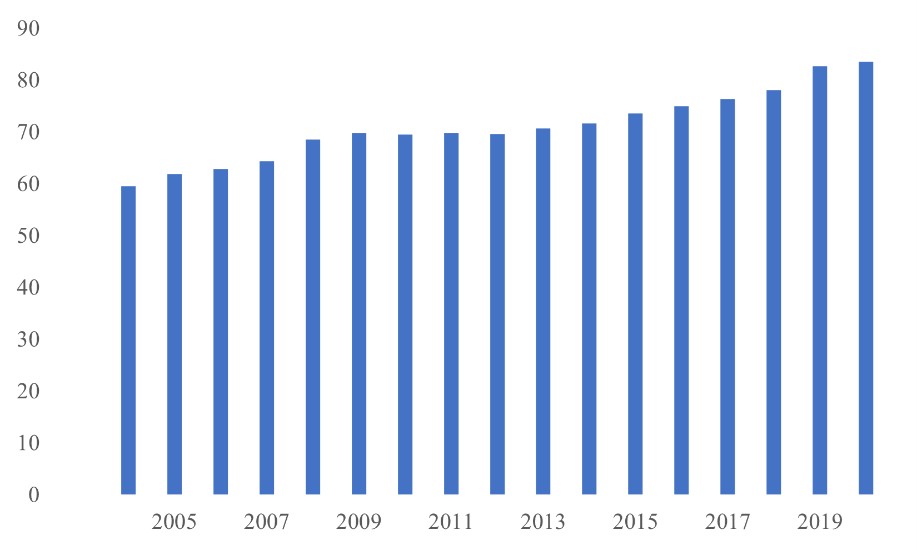


S1 Fig. The mean of the ease of doing business (EDB) index S2 Figure. The mean of starting business (SB)


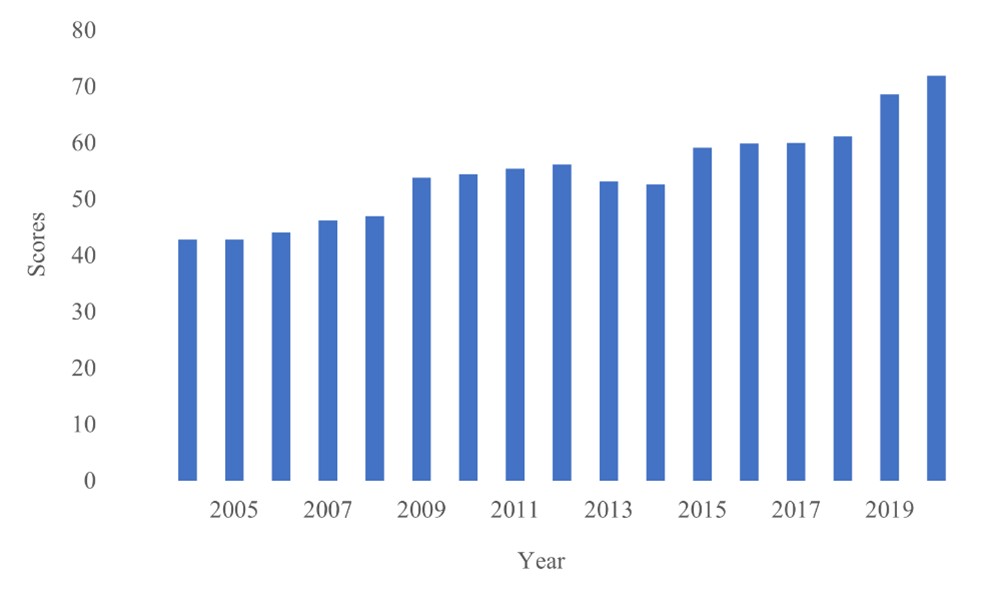

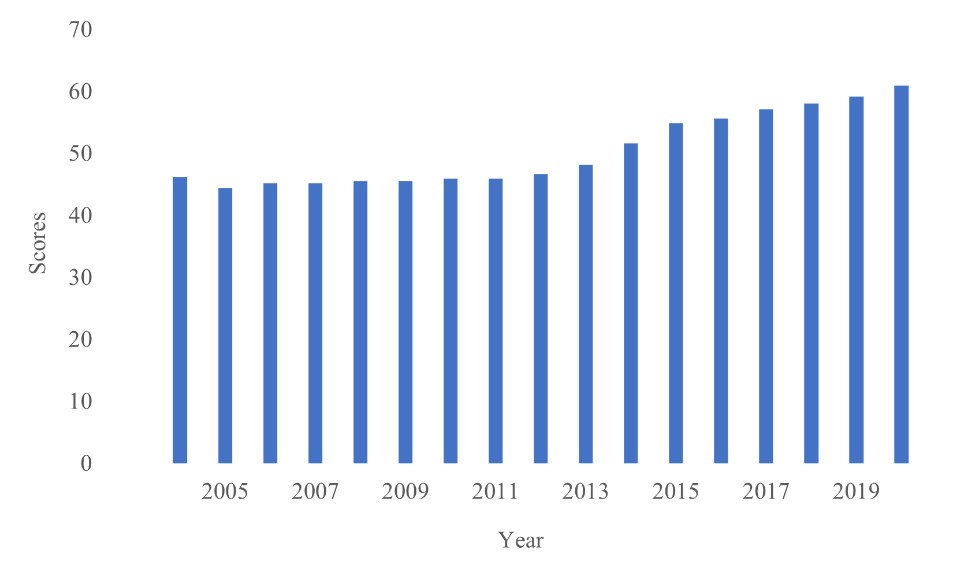


S3 Fig. The mean of dealing with construction permits (DCP) S4 Fig. The mean of dealing with protecting minority investors (PMI)


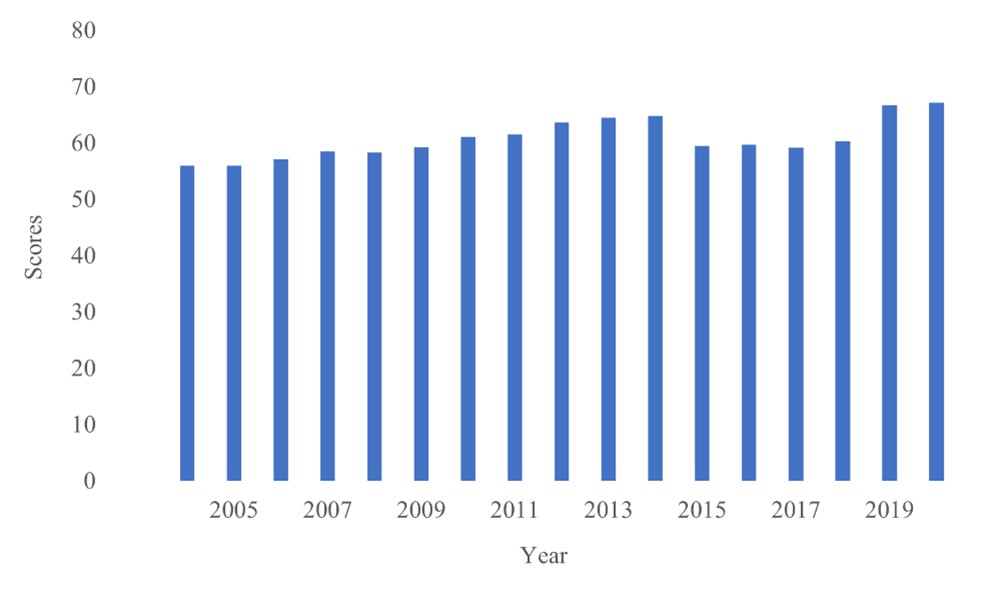

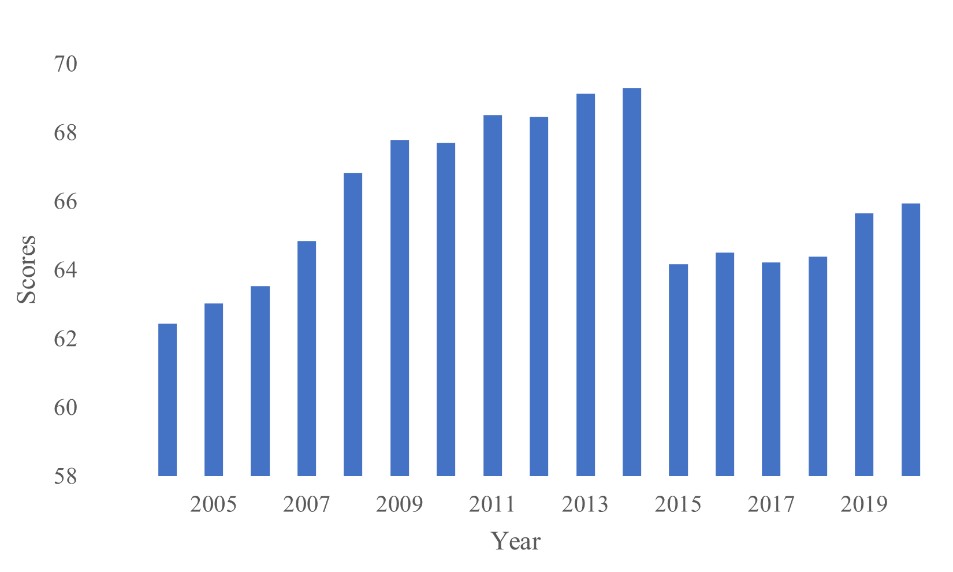


S5 Fig. The mean of trading across borders (TAB) S6 Fig. The mean of dealing with registering property (RP)


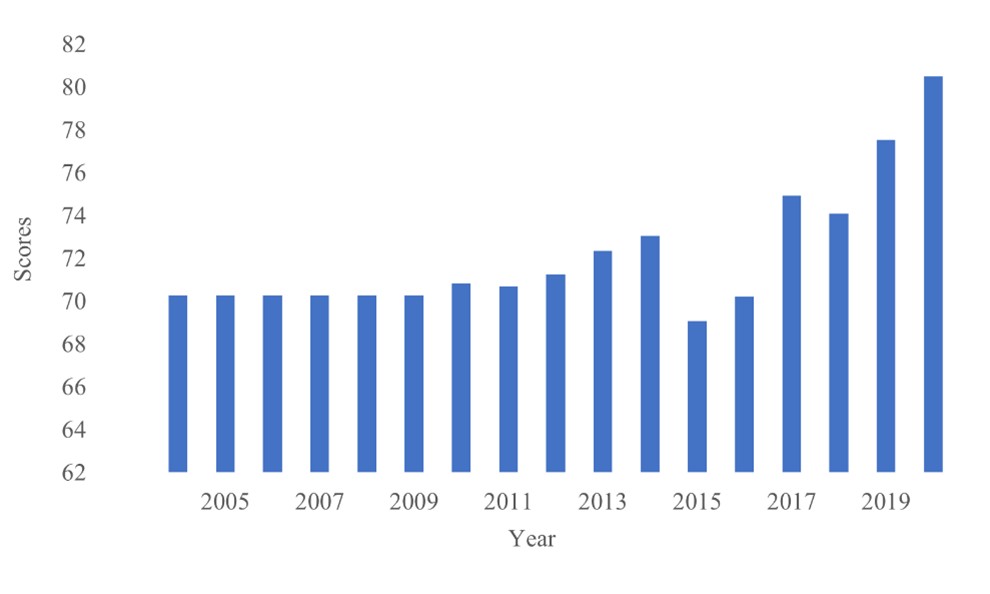

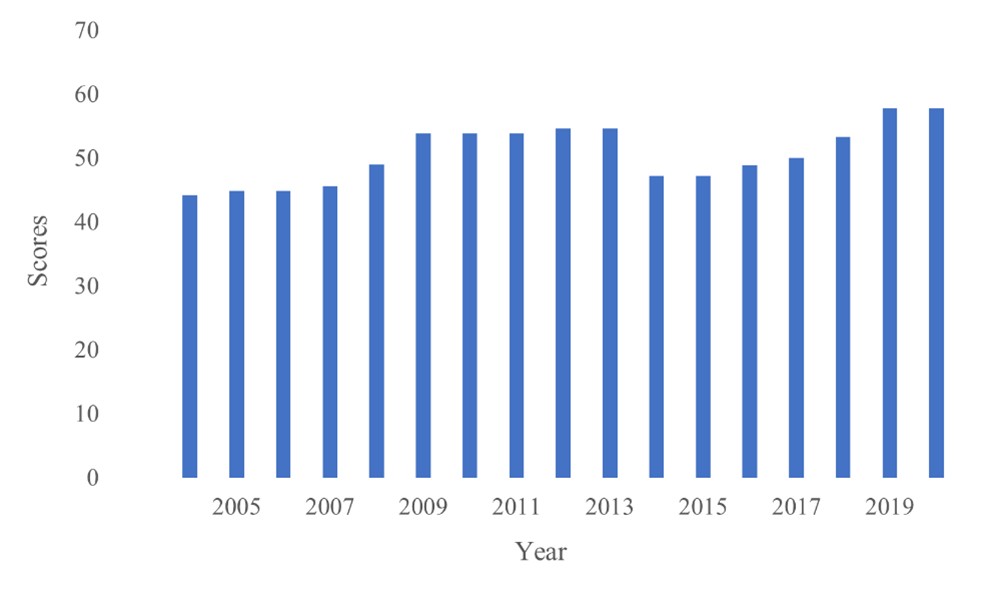


S7 Fig. The mean of dealing with getting electricity (GEL) S8 Fig. The mean of dealing with getting credit (GC)


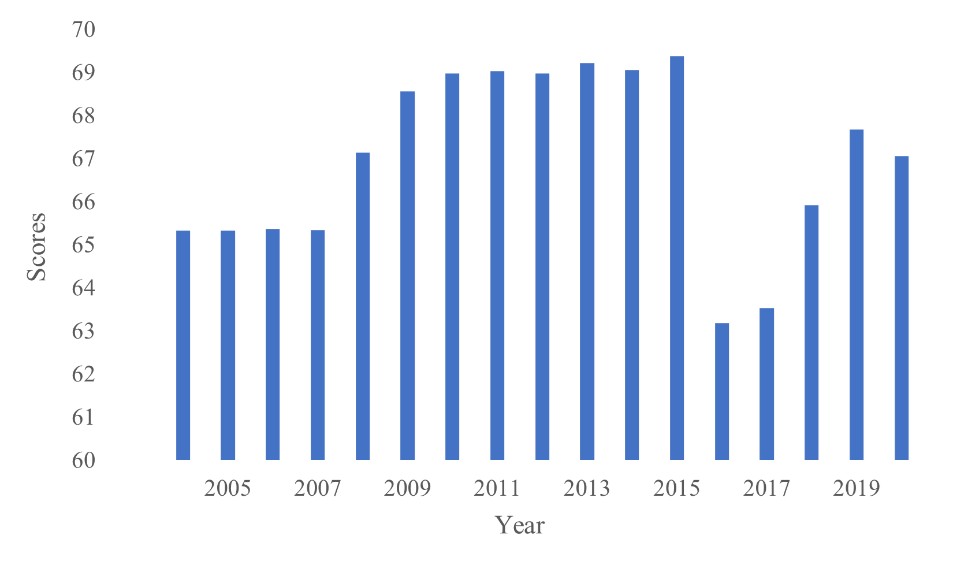

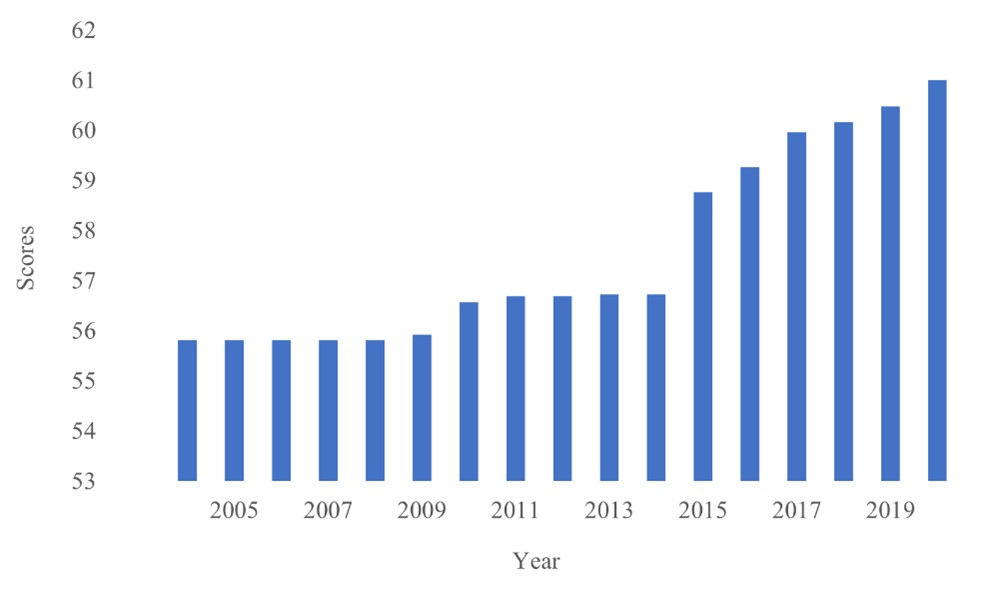


S9 Fig. The mean of dealing with paying taxes (PT) S10 Fig. The mean of enforcing contracts (EC)


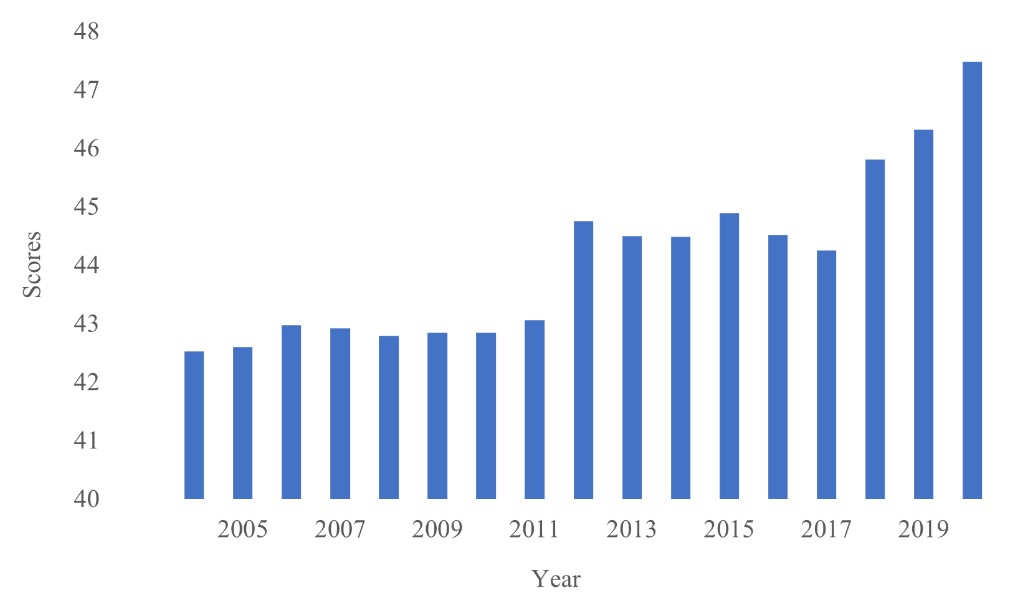


Fig S11. The mean of resolving insolvency (RI)
